# A Review on Phytochemicals of the Genus *Maytenus* and Their Bioactive Studies

**DOI:** 10.3390/molecules26154563

**Published:** 2021-07-28

**Authors:** Yuan-Yuan Huang, Lu Chen, Guo-Xu Ma, Xu-Dong Xu, Xue-Gong Jia, Fu-Sheng Deng, Xue-Jian Li, Jing-Quan Yuan

**Affiliations:** 1 Scientific Experimental Center of Guangxi University of Chinese Medicine, Nanning 530200, China; yuanyhuang@yeah.net (Y.-Y.H.); jxg101313@163.com (X.-G.J.); dengfusheng9527@163.com (F.-S.D.); 2 School of Chemistry and Materials, Nanning Normal University, Nanning 530001, China; 3 Research Department of Guangxi Botanical Garden of Medicinal Plants, Nanning 530023, China; chenlulu982@hotmail.com; 4 Institute of Medicinal Plant Development, Peking Union Medical College and Chinese Academy of Medical Sciences, Beijing 100193, China; mgxfl8785@163.com (G.-X.M.); xdxu@implad.ac.cn (X.-D.X.)

**Keywords:** *Maytenus*, triterpenoids, sesquiterpenes, alkaloids, synthesis of maytansine

## Abstract

The genus *Maytenus* is a member of the Celastraceae family, of which several species have long been used in traditional medicine. Between 1976 and 2021, nearly 270 new compounds have been isolated and elucidated from the genus *Maytenus*. Among these, maytansine and its homologues are extremely rare in nature. Owing to its unique skeleton and remarkable bioactivities, maytansine has attracted many synthetic endeavors in order to construct its core structure. In this paper, the current status of the past 45 years of research on *Maytenus*, with respect to its chemical and biological activities are discussed. The chemical research includes its structural classification into triterpenoids, sesquiterpenes and alkaloids, along with several chemical synthesis methods of maytansine or maytansine fragments. The biological activity research includes activities, such as anti-tumor, anti-bacterial and anti-inflammatory activities, as well as HIV inhibition, which can provide a theoretical basis for the better development and utilization of the *Maytenus*.

## 1. Introduction

Plants of the genus *Maytenus*, a widely distributed member of the Celastraceae family, include approximately 300 plant species that are spread in tropical and subtropical regions of the world [1]. The genus *Maytenus* is widely used in folk medicines around the world, with the roots, bark and leaves being used for the treatment of cancer, gastric ulcers and arthritis because of their anti-inflammatory, analgesic, antiallergic and antitumor properties [2,3,4,5]. Studies have shown that a diverse group of chemical substances, triterpenoids, sesquiterpenes and alkaloids, are responsible for the various biological activities of the plants in this genus [6]. Among them, the macrolide alkaloid, maytansine, was first isolated from *M. serrata* [7], and was shown to be an anti-tumor agent with a novel structure, having some clinical potential. In a clinical trial, maytansine was shown to have promising anti-tumor activities against lymphocytic leukemia, lymphoma, ovarian cancer, breast cancer and melanomas [8,9]. Owing to its unique skeleton and remarkable bioactivity, maytansine has attracted a lot of interest for the possible reconstruction of its core structure. Many synthetic studies of the partial structure of maytansine have been reported. Furthermore, several friedelane triterpenoids with their aromatized characteristic structures and sesquiterpene pyridine alkaloids have been isolated from the genus *Maytenus*, and these have also showed good anti-tumor [10,11] and anti-bacterial [12] characteristics. The new chemical constituents and biological activities of *Maytenus* are given in this review of work from the past 45 years, as well as several chemical synthesis methods of maytansine or maytansine fragments, with the view of realizing their potential development and utilization in the medical field.

## 2. Chemical Constituents of *Maytenus*

Over the past decades, a large variety of biologically active secondary metabolites have been isolated and identified from the members of the genus *Maytenus*, which include a series of triterpenoids, such as friedelane triterpenoids, lupane triterpenes, oleanane triterpenes, sesquiterpenes and their alkaloids, along with some potent anti-tumor maytansinoids. Many scholars have extensively investigated the species, which belong to the genus *Maytenus*, and they have isolated several novel compounds with a wide variety of structures, which may prove to be useful against different diseases.

### 2.1. Triterpenoids

The genus *Maytenus* is a rich source of triterpenoids. These types of compounds are characteristic components found in this genus. The known triterpenoids from 1995 to 2005 were summarized by Zhang et al. [13] and Pu et al. [14,15]. Since then, several new triterpenoids have been discovered. Therefore, we have attempted to update all the data relating to the new triterpenoids isolated from the genus *Maytenus* from 1976 to 2021.

#### 2.1.1. Friedelane Friterpenoids

Friedelane triterpenoids are important characteristic components of the Celastraceae family. Moreover, they are endowed with novel chemical diversity and possess a broad spectrum of biological activities. The friedelane triterpenoids are pentacyclic triterpenes composed of 30 carbons, which are converted from oleanolic acid by methyl shifts. In the five six-membered rings, the A/B, B/C and C/D rings are all *trans* and the D/E rings are mostly *cis* (i.e., H-18*β*). There is one *β*-CH_3_ substitution at each of the C-4, C-5, C-9, C-14 and C-17 positions. The C-3 position is often substituted with a hydroxyl group, although sometimes the hydroxyl group is oxidized to a carbonyl group.

The compound pristimerin (**1**) was isolated from *M. chuchuhuasca* [16]. A new nortriterpene quinone methide, 15*α*-hydroxy-21-keto-pristimerine (**2**), has been obtained from the root bark of *M. catingarum* [17]. Fourteen compounds, including 2,3,22*β*-trihydroxy-24,29-dinor-1,3,5(10), 7-friedelatetraene-6,21-dione-23-al (**3**), 2,22*β*-dihydroxyl-3-methoxy-24,29-dinor-1,3,5(10), 7-friedelatetraene-6,21-dione (**4**), 2,3,22*β*-triihydroxy-23,24,29-trinor-1,3,5(10), 7-friedelatetr aene-6,21-dione (**5**), 2,22*β*-dihydroxyl-3-methoxy-24,29-dinor-1,3,5(10), 7-friedelatetraene-6,21-dione (**6**), 2,3,22*β*-trihydroxy-24,29-dinor-1,3,5(10)-friedelatetraene-6,21-dione (**7**), 2,15*α*,22*β*-trihydroxy-3-methoxy-24,29-dinor-1,3,5(10)-friedelatriene-21-one (**8**), 3,22*β*-dihydroxy-24,29-dinor-l(10)-3,5-friedelatriene-2,7,21-trione (**9**), 3,22*β*-dihydroxy-24,29-dinor-l(10), 3,5-friedelatriene-21-one (**10**), 2,3,22*β*-trihydroxy-24,29-dinor-25(9→8)-1,3,5(10), 7-friedelatetraene-21-one-23-al (**11**), 23-oxo-iso-tingenone (**12**), (8*S*)-7,8-dihydro-7-oxo-tingenoe (**13**), (7*S*,8*S*)-7-hydroxy-7,8-dihydro-tingenone (**14**), (8*S*)-7,8-dihydro-6-oxo-tingenol (**15**) and 23-nor-6-oxo-tingenol (**16**) were isolated from the roots of *M. amazonica* [18,19]. Compounds 28-hydroxy-friedelane-1,3-dione (**17**) and macrocarpins A–D (**18**–**21**) were obtained from the roots of *M. macrocarpa* [20,21], while maytenfolone (**22**) has been isolated from *M. diversifolia* [22]. Three compounds 6-oxo-iguesterol (**23**), 6-oxo-tingenol (**24**) and 3-*O*-methoxy-6-oxo-tingenol (**25**) have been obtained from the root bark of *M. canariensis* [12]. Four new triterpenes blepharotriol (**26**), 6-deoxoblepharodol (**27**), isoblepharodol (**28**) and 7-oxo-blepharodol (**29**) were separated from *M. blepharodes* [23].

**Figure 1:**
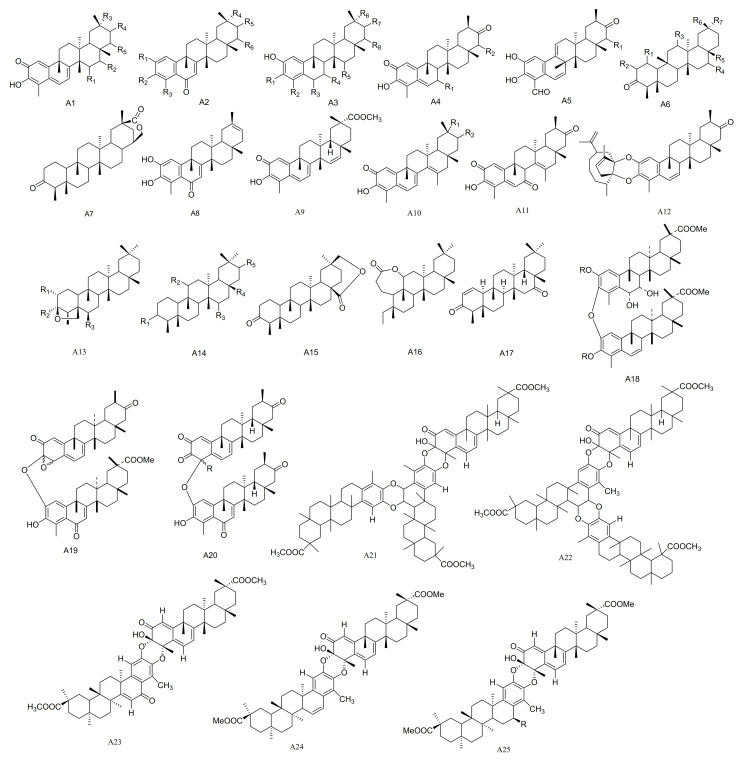
Twenty-five types (A1–A25) of friedelane triterpenoids skeletons.

Compounds 15α-hydroxy-tingenone (**30**), 15-dehydro-pristimerin (**31**), vitideasin (**32**) and 20*β*-hydroxy-scutione (**33**) were separated from the roots of *M. vitis-idaea* [24]. Six new compounds, including 7-oxo-7, 8-dihydro-scutione (**34**), 6,23-dioxo-7,8-dihydro-pristimerol-23-oic Acid (**35**), 23-nor-blepharodol (**36**), 3-methoxy-6-oxo-tingenol-23-oic Acid (**37**), retusonine (**38**) and 21-Oxopristimerine (**39**) were isolated from the root bark of *M. retusa* [25]. A new compound 3-*O*-Methyl-6-oxo-pristimerol (**40**) has been isolated from the hexane/Et_2_O 1:1 extract of the root bark of *M. chubutensis* [26]. Compounds 3*β*,24-epoxy-2*α*,3*α*-dihydroxy-D:A-friedooleanan-29-oic acid methyl ester (**41**), 2*α*-acetoxy-3*β*,24-epoxy-3*α*-hydroxy-D:A-friedooleanan-29-oic acid methyl ester (**42**), 3*α*-hydroxy-D:A-friedooleanan-28-oic acid (**43**) and 3-oxo-D:A-friedooleanan-28,30-olide (**44**) were obtained from the root bark of *M. jelskii* [27]. Compounds 3*β*,11*β*-dihydroxyfriedelane (**45**) and 3,4-seco-friedelan-3,11*β*-olide (**46**) have been obtained from the hexane extracts of the leaves of *M. robusta* [28], while (16*β*)-16-hydroxy-pristimerin (**47**) was from *M. salicifolia* [29]. A new triterpenoid, 12,16-dihydroxyfriedelan-3-one (**48**), was isolated from an ethyl acetate extract of *M.* *oblongata* [30]. Compounds 3*β*,24*β*-epoxy-29-methoxy-2*α*,3*α*,6*α*-trihydroxy-D:A-friedelane (**49**) and 3*β*,24*β*-epoxy-29-methoxy-2*α*,3*α*,6*α*-triacetoxy-D:A-friedelane (**49a**) were obtained from the root bark extracts of *M. cuzcoina* [31]. Three new pentacyclic triterpenoids, friedel-1-en-3,16-dione (**50**), 1*α*,29-dihydroxyfriedelan-3-one (**51**) and 16*β*,28,29-trihydroxyfriedelan-3-one (**52**) have been separated from *M. robusta* [32]. Dispemroquinone (**53**) was isolated from *M. dispermus* [33]. A new norquinonemethide triterpene with a netzahualcoyene type skeleton, scutione (**54**), was isolated from the root bark of *M. scutioides* [34]. Compounds zeylasterone (**55**) and demethylzeylasterone (**56**) were obtained from *M. blepharodes* [35], and compound 3,15-dioxo-21*α*-hydroxy friedelane (**57**) was isolated from the methanol extracts of *M. robusta* [36]. Maytenfoliol (**58**) was separated from *M. diversifolium* [37]. Four new cytotoxic triterpenoid dimers, including cangorosin A (**59**), atropcangorosin A (**60**), dihydroatropcangorosin A (**61**) and cangorosin B (**62**) were obtained from the extracts of *M. ilicifolia* [38]. Two new triterpenes, umbellatin *α* (**63**) and umbeilatin *β* (**64**), have been separated from *M. umbellata* [39]. Two novel trimer triscutins, A and B (**65**–**66**), have been isolated from extracts of the root bark of *M. scutioides* [40]. Four new triterpene dimers, xuxuarine E*α* (**67**), scutionin *α*B (**68**), 6′,7′-dihydro-scutionin *α*B (**69**) and 6′*β*-methoxy-6′,7′dihydro-scutionin *α*B (**70**), have been isolated from the extracts of the roots of *M. blepharodes* and *M. magellanica* [41,42] (Table 1 and Figure 1).

#### 2.1.2. Lupane Triterpenes

**Figure 2:**
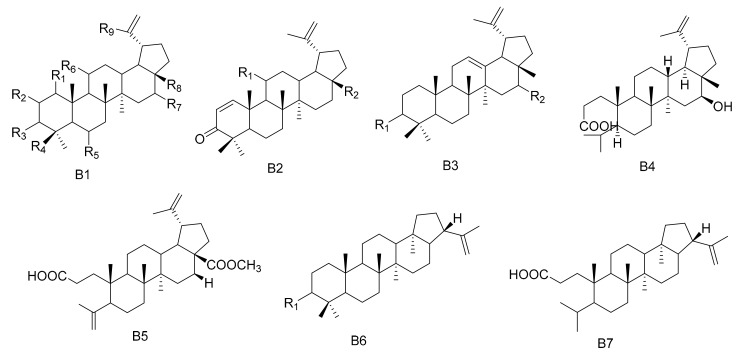
Seven types (B1–B7) of lupane triterpenes skeletons.

Lupane triterpenes are characterized by the combination of C-21 and C-19, clustered into a five-membered carbocyclic E ring. There is an isopropyl group substituted at the 19th position of the E ring with an *α* configuration, as well as a double bond at the C-20(29) position. The rings of the A/B, B/C, C/D and D/E types are all *trans*. The new triterpenes 3*β*,28,30-Lup-20(29)-ene triol (**71**) and 28,30-Dihyroxylup-20(29)-ene-3-one (**72**) were obtained from *M. canariensis* [43], while compound maytefolin A (**73**) was isolated from the leaves of a Brazilian medicinal plant, *M. ilicifolia* [44]. 3-oxo-lup-20(29)-en-30-al (**74**), 30-hydroxylup-20(29)-en-3-one (**75**), (11*α*)-11-hydroxylup-20(29)-en-3-one (**76**) and (3*β*)-lup-20(30)-ene-3,29-diol (**77**) have been obtained from the hexane extracts of the stems and branches of *M. imbricate* [45]. Compounds 11*α*-hydroxy-*epi*-betuin (**78**), 6*β*-hydroxybetulin (**79**), 24-hydroxybetulin (**80**), rigidenol-28-aldehyde (**81**) and 28-hydroxyglochidone (**82**) have been isolated from *M. cuzcoina* and *M. chiapensis* [46]. Compounds 11*α*-hydroxy-glochidone (**83**), 3-*epi-*nepeticin (**84**) and 3-*epi-*calenduladiol (**85**) were separated from the root barks of *M. cuzcoina* and the leaves of *M. chiapensis* [47]. Four new triterpenes, including 3*α*,16*β*,28-Trihydroxylup-20(29)-ene (**86**), 3*α*,16*β*-dihydroxylup-12-ene (**87**), 3*β*,16*β*-dihydroxylup-12-ene (**88**) and 16*β*-3,4-Secolup-20(29)-en-3-oic acid (**89**), were obtained from the aerial parts of *M. apurimacensis* [48], while compound 3-(*E*)-*β*-coumaroylnepeticin (**90**) was isolated from *M. retusa* [25]. Compound 3,4-*seco*-lupa-4(23): 20(29)-diene-3,28-dioic acid 28-methyl ester (**91**) has been separated from the hexane/Et_2_O 1:1 extracts of the root barks of *M. magellanica* [26]. 1*β*-Hydroxy-3*β*-caffeate lup-20(29)-ene (**92**) was isolated from the roots of *M. apurimacensis* [49]. Compounds 3-oxo-21*β*-*H*-hop-22(29)-ene (**93**), 3*β*-hydroxy-21*β*-*H*-hop-22(29)-ene (**94**) and 3,4-seco-21*β*-*H*-hop-22(29)-en-3-oic acid (**95**) were isolated from the leaves of *M. robusta* [28] (Table 2 and Figure 2).

#### 2.1.3. Oleanane Triterpenes

**Figure 3:**
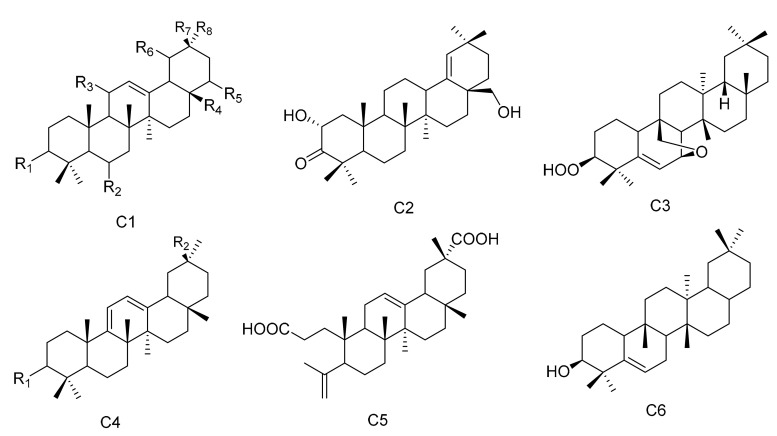
Six types (C1–C6) of oleanane triterpenes skeletons.

Oleanane triterpenes are widely distributed in the plant kingdom. The configuration of the rings is A/B, B/C and C/D, and they are all of the *trans* configuration, while the D/E ring is *cis*. There are eight methyl groups on the core nuclei, and the methyl groups at positions C-10, C-8 and C-17 are all *β* configuration. The methyl group at the C-14 position is *α* configuration, while the C-4 and C-20 positions each have two methyl groups. There may also be other substituents present in the molecule. Two new oleanane triterpenes, 3*β*,19*α*-dihydroxyolean-12-en-29-oic acid (**96**) and 3*α*,19*α*-dihydroxyolean-12-en-29-oic acid (**97**), were obtained from *M. austyoyunnanensis* [14]. Compound 3-oxo-11*α*-methoxyolean-12-ene (**98**) was obtained from the extracts of the roots of *M. spinosa* [24], while 22*α*-hydroxy-29-methoxy-3*β*-tetradecanoate-olean-12-ene (**99**) was separated from the root bark extracts of *M. cuzcoina* [31]. The new compound maytefolin B (**100**) was separated from the leaves of a Brazilian medicinal plant, *M. ilicifolia* [44]. One new triterpene, 3*β*-peroxy-7*β*,25-epoxy-D:B-friedoolean-5-ene (**101**), was separated from the aerial parts of *M. apurimacensis* [48]. Compounds krukovines A (28-hydroxyolean-12-ene-3,11-dione) (**102**) and krukovines C (6*β*,28-dihydroxyolean-12-ene-3,11-dione) (**103**) have been obtained from a South American medicinal plant known as “chuchuhuasi” (*M. krukovii*) [50]. The aerial parts of *M. undata* yielded four new 12-oleanene and 3,4-*seco*-12-oleanene triterpene acids, namely, 3-oxo-11*α*-methoxyolean-12-ene-30-oic acid (**104**), 3-oxo-11*α*-hydroxyolean-12-ene-30-oic acid (**105**), 3-oxo-olean-9(11), 12-diene-30-oic acid (**106**) and 3,4-*seco*-olean-4(23), 12-diene-3,29-dioic acid (**107**) [51], while 3*α*-22*β*-dihydroxyolean-12-en-29-oicacid (**108**) was obtained from the methanol extracts of the barks of *M. laevis* [52]. Compound olean-9(11):12-dien-3*β*-ol (**109**) was isolated from the roots of *M. acanthophylla* [53] and compound 3*β*-hydroxy-D:B-friedo-olean-5-ene (**110**) was isolated from *M. salicifolia* Reissek [54]. Compound 19*α*-hydroxy-3-olean-12-en-29-oic acid (**111**) was isolated from *M. austyoyunnanensis* [55] (Table 3 and Figure 3).

#### 2.1.4. Other Triterpenes

**Figure 4:**
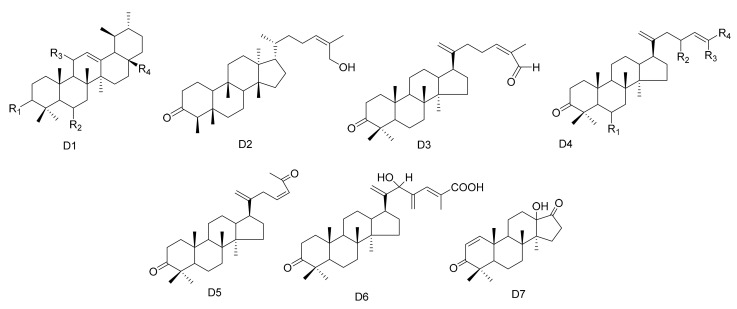
Seven types (D1–D7) of other triterpenes skeletons.

In addition to the above, other types of triterpene compounds have also been isolated from *Maytenus*. These include triterpene dimers, ursane triterpenes and dammarane triterpenes. The compound 3-Oxo-methoxyurs-12-ene (**112**) was isolated from *M.* *spinosa* [24]. Three ursane triterpenes, krukovines B, D and E (**113**–**115**), were obtained from *M. krukovii* [50]. Compound maytefolin C (**116**) has been isolated from the leaves of *M. ilicifolia* [44], while 28-hydroxy-12-ursene-3*β*-yl-caffeate (uvaol-3-caffeate) (**117**) has been isolated from the methanol extracts of the barks of *M. laevis* [52]. An ursane triterpene 3*β*-stearyloxy-urs-12-ene (**118**) was obtained from *M. salicifolia* [56]. The stem bark exudates of *M. macrocarpa* yielded ten dammarane triterpenes, namely, 24-(*E*)-3-oxo-dammara-20,24-dien-26-al (**119**), 24-(*Z*)-3-oxo-dammara-20,24-dien-26-al (**120**), 24-(*E*)-3-oxo-dammara-20,24-dien-26-ol (**121**), 24-(*E*)-3-oxo-dammara-23-*α*-hydroxy-20,24-dien-26-al (**122**), 24-(*E*)-3-oxo-dammara-23-*β*-hydroxy-20,24-dien-26-al (**123**), 24-(*E*)-3-oxo-dammara-6-*β*-hydroxy-20, 24-dien-26-al (**124**), 24-(*E*)-3-oxo-dammara-6-*β*-hydroxy-20,24-dien-26-ol (**125**), 23-(*Z*)-3, 25-dioxo-25-nor-dammara-20,24-diene (**126**), 24-(*E*)-3-oxo-23-methylene-dammara-20,24-dien-26-oico (**127**), 24(*Z*)-3-oxodammara20(21),24-dien-27-oic acid (**128**) and octa-nor-13-hydroxydammara-1-en-3,17-dione (**129**). This was in 1997, and it was the first time that dammrane triterpenes were isolated from Celastraceae [57,58] (Table 4 and Figure 4).

### 2.2. Sesqiterpenoids

The most widespread and characteristic metabolites isolated from the Celastraceae family are a large group of unusual and highly oxygenated sesquiterpenoids, based on the [5,11-epoxy-5*β*,10*α*-eduesman-4(14)-ene] skeleton known as dihydro-*β*-agarofum. Sesquiterpenes have multiple substitution sites in their structure, and common substituents include -OH, -OAc, -Ofu, -Obz and -Onic, which is due to the diversification of their positions and types. There is a high probability that there are several new compounds from this group that still need to be discovered [59].

Two sesquiterpene polyesters with new polyhydroxy skeletons, 1*α*,9*α*-dibenzoyloxy-6*β*,8*α*,15-triacetoxy-4*β*-hydroxy-dihydro-*β*-agarofurane (**130**) and 1*α*,9*α*-dibenzoyloxy-2*α*,6*β*,8*α*,15-tetracetoxy-4*β*-hydroxydihydro-*β*-agrofurane (**131**), were isolated from the aerial portions of *M. canariensis* [60]. Compounds 6*β*,8*β*-15-triacetoxy-1*α*,9*α*-dibenzoyloxy-4*β*-hydroxy-*β*-dihydroagarofuran (**132**), 1*α*,6*β*,8*β*,15-tetraacetoxy-9*α*-benzoyloxy-4*β*-hydroxy-*β*-dihydroagarofuran (**133**) and (1*S*,4*S*,6*R*,7*R*,8*R*,9*R*)-1,6,15-triacetoxy-8,9-dibenzoyloxy-4*β*-hydroxy-*β*-dihydroagarofuran (**134**) were isolated from the aerial parts of *M. macrocarpa* [61]. Compounds (1*R*,2*S*,4*S*,5*S*,6*R*,7*R*,9*S*,10*S*)-6,15-diacetoxy-1,2,9-tribenzoyloxy-4-hrdroxy-8-oxo-dihydro-*β*-agarofuran (**135**) and 9*β*-cinnamoyloxy-2*β*,3*β*-diacetoxy-6*β*-hydroxy-l*α*-nicotinoyloxydihidro-*β*-agarofuran (**136**) were separated from *M. blepharodes* [41]. Eight sesquiterpenoids, including 1*α*-acetoxy-2*α*,6*β*,9*β*-trtifuroyloxy-4*β*-hydroxy-dihydro-*β*-agarofuran (**137**), 1*α*,2*α*-diacetoxy-6*β*,9*β*-difuroyloxy-4*β*-hydroxy-dihydro-*β*-agarofuran (**138**), 1*α*-acetoxy-6*β*,9*β*-difuroyloxy-2*α*,4*β*-dihydroxy-dihydro-*β*-agarofuran (**139**), 1*α*-acetoxy-2*α*-benzoyloxy-6*β*,9*β*-difuroyloxy-4*β*-dihydro-*β*-agarofuran (**140**), 1*α*-acetoxy-6*β*,9*β*-difuroyloxy-2*α*-propyonyloxy-4*β*-hydroxy-dihydro-*β*-agarofuran (**141**), 1*α*-acetoxy-6*α*,9*β*-difuroyloxy-2*α*-(2)-methylbutyroyloxy-4*β*-hydroxy-dihydro-*β*-agarofuran (**142**), 1*α*,2*α*,15-triacetoxy-6*β*,9*β*-difuroyloxy-4*β*-hydroxy-dihydro-*β*-agarofuran (**143**) and 1*α*,2*α*,15-triacetoxy-6*β*,9*β*-dibenzoyloxy-4*β*-hydroxy-dihydro-*β*-agarofuran (**144**) were obtained from the n-hexane: Et_2_O (1:1) extracts of the fruits of *M. cuzcoina* [59].

The *n*-hexane/Et_2_O (1:1) extracts of the root barks of *M. magellanica* yielded eight new dihydro-*β*-agarofuran sesquiterpenes (**145**–**152**), and the *n*-hexane/Et_2_O (1:1) extracts of the root barks of *M. chubutensis* yielded two more new compounds of this family (**153**–**154**). Their structures were elucidated as (1*R*,2*R*,4*S*,5*R*,7*S*,9*S*,10*R*)-2-acetoxy-1-benzoyloxy-9-cinnamoyloxy-4-hydroxy-dihydro-*β*-agarofuran (**145**), (1*R*,2*S*,3*S*,5*R*,7*R*,9*S*,10*R*)-2-acetoxy-9-benzoyloxy-1-cinnamoyloxy-3-nicotinoyloxy-4-hydroxy-dihydro-*β*-agarofuran (**146**), (1*R*,2*S*,3*S*,4*S*,5*S*,6*R*,7*R*,9*S*,10*R*)-2,6-diacetoxy-1-benzoyloxy-9-cinnamoyloxy-3-nicotinoyloxy-4-hydroxy-dihydro-*β*-agarofuran (**147**), (1*R*,2*S*,3*S*,4*S*,5*S*,6*R*,7*R*,9*S*,10*R*)-2,6-diacetoxy-1,9-dibenzoyloxy-3-nicotinoyloxy-4-hydroxy-dihydro-*β*-agarofuran (**148**), (1*R*,2*S*,3*S*,4*S*,5*R*,7*S*,8*S*,9*R*,10*R*)-2,3-diacetoxy-8,9-dibenzoyloxy-1-nicotinoyloxy-4-hydroxy-dihydro-*β*-agarofuran (**149**), (1*R*,2*S*,4*S*,5*S*,6*R*,7*R*,8*S*,9*R*,10*S*)-6,8-diacetoxy-1,2,9-tribenzoyloxy-4-hydroxy-dihydro-*β*-agarofuran (**150**), (1*R*,2*S*,3*S*,4*S*,5*R*,7*S*,8*S*,9*R*,10*R*)-2,8-diacetoxy-3,9-dibenzoyloxy-1-nicotinoyloxy-4-hydroxy-dihydro-*β*-agarofuran (**151**), (1*R*, 2*S*,4*R*,5*S*,6*R*,7*R*,8*S*,9*R*,10*S*)-6,8-diacetoxy-1,9-dibenzoyloxy-2-nicotinoyloxy-dihydro-*β*-agarofuran (**152**), 1*α*,15-diacetoxy-6*β*,9*β*-dibenzoyloxy-2*α*-nicotinoyloxy-dihydro-*β*-agarofuran (**153**) and 1*α*,15-diacetoxy-6*β*,9*β*-dibenzoyloxy-2*α*-nicotinoyloxy-4*β*-hydroxy-dihydro-*β*-agarofuran (**154**) [62]. Compounds (1*R*,2*S*,4*S*,5*S*,6*R*,7*R*,9*S*,10*S*)-1,2,6,9,15-pentaacetoxy-4-hydroxy-8-oxo-dihydro-*β*-agarofuran (**155**), (1*R*,2*S*,4*S*,5*S*,6*R*,7*R*,9*S*,10*S*)-1,2,9,15-taacetoxy-4,6-dihydroxy-8-oxo-dihydro-*β*-agarofuran (**156**), (1*R*,2*S*,4*S*,5*S*,6*R*,7*R*,9*S*,10*S*)-1,9,15-triacetoxy-2,4,6-trihydroxy-8-oxo-dihydro-*β*-agarofuran (**157**), (1*R*,2*S*,3*S*,4*S*,5*S*,6*R*,7*R*,9*S*,10*S*)-1,2,3,6,9,12,15-heptaacetoxy-4-hydroxy-8-oxo-dihydro-*β*-agarofuran (**158**) and 1*α*,2*α*,3*β*,6*β*,8*α*,9*α*,12,15-octaacetoxy-4*β*-hydroxy-dihydro-*β*-agarofuran (**159**) were isolated from the leaves of *M. chiapensis* [63]. In addition, (1*S*,4*S*,5*S*,6*R*,7*R*,8*S*,9*R*,10*R*)-8-acetoxy-1,9-dibenzoyloxy-6-nicotynoyloxy-dihydro-*β*-agarofuran (**160**) and (1*S*,4*R*,5*R*,6*R*,7*R*,8*S*,9*R*,10*R*)-8-acetoxy-1,9-dibenzoyloxy-4-hydroxy-nicotynoyloxy-dihydro-*β*-agarofuran (**161**) have been isolated from the roots of *M. apurimacensis* [49].

Thirteen sesquiterpenes, including (1*R*,2*S*,4*S*,5*S*,6*R*,7*R*,9*S*,10*R*)-115-diacetoxy-2,6-dibenzoyloxy-9-(3-furoyloxy)-4-hydroxy-dihydro-*β*-agarofuran (**162**), (1*R*,2*S*,4*S*,5*S*,6*R*,7*R*,9*S*,10*R*)-1,2,15-triacetoxy-6-benzoyloxy-9-(3-furoyloxy)-4-hydroxy-dihydro-*β*-agarofuran (**163**), (1*R*,2*S*,4*S*,5*S*,6*R*,7*R*,9*S*,10*R*)-1,15-diacetoxy-6-benzoyloxy-9-(3-furoyloxy)-2,4-dihydroxy-dihydro-*β*-agarofuran (**164**), (1*R*,2*S*,4*S*,5*S*,6*R*,7*R*,9*S*,10*R*)-1,15-diacetoxy-6,9-dibenzoyloxy-2,4-hydroxy-dihydro-*β*-agarofuran (**165**), (1*R*,2*S*,4*S*,5*S*,6*R*,7*R*,9*S*,10*R*)-1,2,6,15-tetracetoxy-9-(3-furoyloxy)-4-hydroxy-dihydro-*β*-agarofuran (**166**), (1*R*,2*S*,4*S*,5*S*,6*R*,7*R*,9*S*,10*R*)-1-Acetoxy-2,6-dibenzoyloxy-9-(3-furoyloxy)-4-hydroxy-dihydro-*β*-agarofuran (**167**), (1*S*,2*S*,3*S*,4*S*,5*R*,7*R*,9*S*,10*R*)-2,3-diacetoxy-9-benzoyloxy-1-(3-furoyloxy)-4-hydroxy-dihydro-*β*-agarofuran (**168**), (1*S*,2*R*,4*S*,5*R*,7*R*,9*S*,10*R*)-2-acetoxy-9-benzoyloxy-1-(3-furoyloxy)-4-hydroxy-dihydro-*β*-agarofuran (**169**), (1*S*,2*R*,4*S*,5*R*,7*R*,9*S*,10*R*)-2-Acetoxy-1,9-di-(3-furoyloxy)-4-hydroxy-dihydro-*β*-agarofuran (**170**), (1*S*,2*R*,4*S*,5*R*,7*R*,9*S*,10*R*)-2-Acetoxy-9-trans-cynamoiloxy-1-(3-furoyloxy)-4-hydroxy-dihydro-*β*-agarofuran (**171**), (1*S*,4*S*,5*R*,7*R*,9*S*,10*S*)-9-Benzoyloxy-1-(3-furoyloxy)-4-hydroxy-dihydro-*β*-agarofuran (**172**), (1*S*,2*R*,3*R*,4*R*,5*S*,7*R*,9*S*,10*R*)-2,3-diacetoxy-9-benzoyloxy-1-(3-furoyloxy)-dihydro-*β*-agarofuran (**173**) and (1*S*,2*R*,4*R*,5*S*,7*R*,9*S*,10*R*)-2-Acetoxy-9-benzoyloxy-1-(3-furoyloxy)-dihydro-*β*-agarofuran (**174**) have been isolated from the hexanee-Et_2_O extracts of the fruits of *M. jelskii* [64]. Nine new *β*-dihydroagarofurans, 1*α*2*α*,9*β*,15-tetracetoxy-8*β*-benzoyloxy-*β*-dihydroagarofuran (**175**), 1*α*-benzoyloxy-2*α*,6*β*,8*α*-triacetoxy-9*α*-methyllbutyroyloxy-*β*-dihydroagarofuran (**176**), 1*α*,6*β*-diacetoxy-2*α*,8*α*,9*α*-tribenzoyloxy-*β*-dihydroagarofuran (**177**), 1*α*-benzoyloxy-2*α*,6*β*,8*α*,9*α*-tetraacetoxy-*β*-dihydroagarofuran (**178**), 1*α*,6*β*,8*α*-triacetoxy-9*α*-benzoyloxy-2*α*-hydroxy-*β*-dihydroagarofuran (**179**), (1*R*,2*S*,4*R*,5*S*,6*R*,7*R*,8*R*,9*S*,10*S*)-1,6-diacetoxy-8,9-dibenzoyloxy-2-h ydroxy-*β*-dihydroagarofuran (**180**), 1*α*,6*β*,15-triacetoxy-8*α*-methylbutyroyloxy-9*α*-benzoyloxy-2*α*-hydroxy-*β*-dihydroagaro-furan (**181**), 1*α*,6*β*,15-triacetoxy-8*α*,9*α*-dibenzoyloxy-2*α*-hydroxy-*β*-dihydroagarofuran (**182**) and 1*α*,6*β*,8*β*,15-tetracetoxy-2*α*-hydroxy-9*α*-benzoyloxy-*β*-dihydroagarofuran (**183**), were isolated from the leaves of *M. spinosa* [65]. Five new compounds, chiapens A–E (**184**–**188**), were isolated from *M. chiapensis* [66].

**Figure 5:**
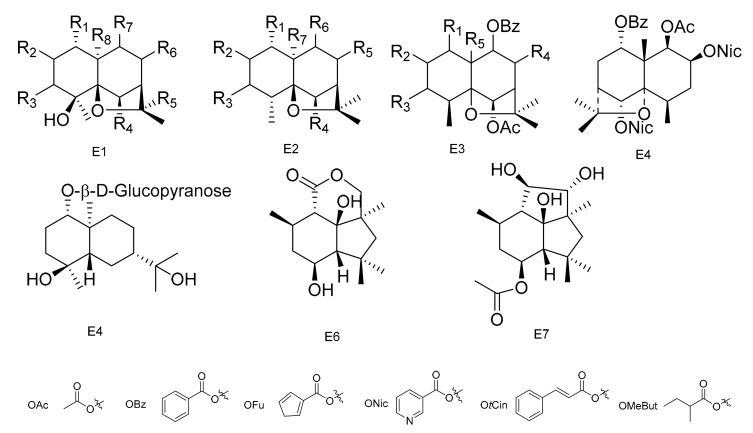
Seven types (E1–E7) of sesqiterpenoids skeletons.

Compounds 1*α*,6*β*-diacetoxy-8*α*-hydroxy-9*β*-furoyloxy-*β*-agarofuran (**189**), 1*α*-acetoxy-6*β*,8*α*-dihydroxy-9*β*-furoyloxy-*β*-agarofuran (**190**), 1*α*-benzoyloxy-2*α*,3*β*,6*β*,9*β*,14-pentaacetoxy-8-oxo-*β*-agarofuan (**191**) and 1*α*-furoyloxy-2*α*,3*β*,6*β*,9*β*,14-pentaacetoxy-8-oxo-*β*-agarofuan (**192**) were obtained from an extract of the seeds of *M. boaria* [67]. Bilocularins A−I (**193**–**201**) were isolated from *M. bilocularis*. In addition, bilocularins D–F are the first examples of dihydro-b-agarofurans, which bear a hydroxyacetate group [68,69]. Compounds (1*S*,4*S*,5*S*,6*R*,7*R*,8*R*,9*R*,10*S*)-6-acetoxy-4,9,10-trihydroxy-2,2,5*a*,9-tetramethyloctahydro-2*H*-3,9*a*-methanobenzo[*b*]oxepin-5-yl furan-3-carboxylate (**202**), (1*S*,4*S*,5*S*,6*R*,7*R*,8*R*,9*R*,10*S*)-6-acetoxy-4,9-dihydroxy-2,2,5*a*,9-tetramethyloctahydro-2*H*-3,9a-methanobenzo[*b*]oxepine-5,10-diyl bis(furan-3-carboxylate) (**203**), (1*S*,4*S*,5*S*,6*R*,7*R*,9*S*,10*S*)-6-acetoxy-9-hydroxy-2,2,5*a*,9-tetramethyloctahydro-2*H*-3,9*a*-methanobenzo[*b*]oxepine-5, 10-diyl bis(furan-3-carboxylate) (**204**) and (1*S*,4*S*,5*S*,6*R*,7*R*,9*S*, 10*S*)-6-acetoxy-10-(benzoyloxy)-9-hydroxy-2,2,5*a*,9-tetramethyloctahydro-2*H*-3,9*a*-methanobenzo[*b*]-oxepin-5-yl furan-3-carboxylate (**205**) were isolated from the seeds of *M. boaria* [70,71]. Compounds 2*β*,6*β*-diacetoxy-1*α*,9*β*-dibenzoyl-3*β*-hydroxy-dihydro-*β*-agarofuran (**206**), 1*α*,2*α*,6*β*,8*α*-tetraacetoxy-9*β*-benzoyl-15-hydroxy-dihydro-*β*-agarofuran (**207**) and 1*α*,2*α*,6*β*,8*α*,15-pentaacetoxy-9*β*-benzoyl-dihydro-*β*-agarofuran (**208**) have been separated from *M. boaria* [72]. 1*β*-acetoxy-9*α*-benzoyloxy-2*β*,6*α*-dinicotinoyloxy-*β*-dihydroagarofuran (**209**) was obtained from the anti-microbially active ethanol extracts of *M. heterophylla* [73]. In addition, an eudesmane glucoside, boarioside (**210**), has been isolated from *M. boaria* [74]. Compounds 4-deacetyl-10-oxo-dihydrobotrydial (**211**) and 4*β*-acetoxy-9*β*,10*β*,15*α*-trihydroxyp robotrydial (**212**) were obtained from solid cultures of an endocytic fungal strain, *Phomopsis* species Lz42, cultivated on *M. hookeri* [75] (Table 5 and Figure 5).

### 2.3. Alkaloids

#### 2.3.1. Sesquiterpene Pyridine Alkaloids

Among the naturally occurring nitrogen containing compounds, the pyridine alkaloids constitute an important group, and these are relatively rare natural products. The Celastraceae family is a rich source of sesquiterpene pyridine alkaloids. These compounds are endowed with a novel type of chemical diversity, and have complicated stereo-chemistries. They also possess a broad spectrum of biological activities, such as having immunosuppressive and anti-tumor properties. The vast majority of macrolide sesquiterpene pyridine alkaloids, from the genus *Maytenus*, are based on the [5,11-epoxy-5*β*,10*a*-eduesman-4(14)-ene] skeleton known as dihydro-*β*-agarofum. These compounds are characterized by a pyridine dicarboxylic acid macrocyclic bridge (such as evoninic, wilfordic and hydroxywilfordic acids), linked via two ester moieties at the C-3 and C-15 positions [65,76]. Many of these alkaloids have been isolated by organic chemists over recent years. Below we summarize their information, including the names of compounds, their original plant source as well as their structures.

**Figure 6:**
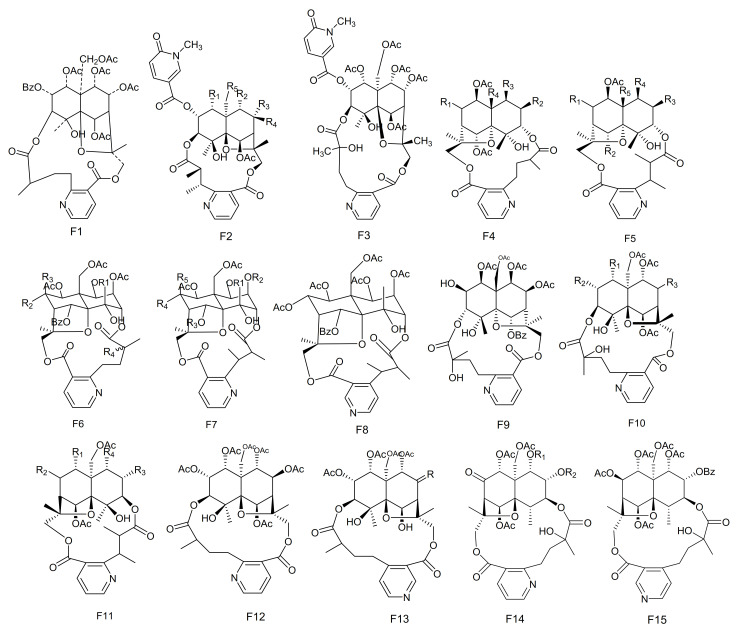
Fifteen types (F1–F15) of sesquiterpene pyridine alkaloids skeletons.

The potent anti-feedant wilforine (**213**) was isolated from *M. rigida* [77]. Compounds emarginatines A–H (**214–221**) and emarginatinine (**222**) were obtained from *M. emarginata* and the leaves of *M. diversifolia*. [11,22,78,79]. Ebenifoline W-I (**223**), ebenifoline E-I (**224**) and ebenifoline E-II (**225**) were separated from the stem bark methanol extracts of *M. ebenifolia* Reiss [80]. Compounds aquifoliunines E-I-IV (**226–229**) have been obtained from the root barks of *M. aquiJolium*. [81,82], while ilicifoliunines A–B (**230–231**) and mayteine (**232**) were isolated from the root barks of *M. ilicifolia* [83]. Laevisines A (**233**) and B (**234**) have been separated from the CHCl_3_:MeOH (9:1) extracts of the barks of *M. laevis* [84]. Compounds mekongensine (**235**), 7-*epi*-mekongensine (**236**), 1-*O*-benzoyl-1-deacetylmekongensine (**237**), 9′-deacetoxymekongensine (**238**), 1-*O*-benzoyl-1-deacetyl-9′-deacetoxymekongensine (**239**), 7-*epi*-euojaponine A (**240**), 2-*O*-benzoyl-2-deacetylmayteine (**241**) and 7-*epi*-5-*O*-benzoyl-5-deacetylperitassine A (**242**) have been isolated from the roots of *M. mekongensis* [85]. The compound 5-benzoyl-5-deacetylwilforidine (**243**) was isolated from *M. buchananii* (Loes.) R. Wilczek. This appears to be the first sesquiterpene nicotinoyl alkaloid found which was based on hydroxywilfordic acid, with a benzoyl group at C-5 position [86]. Compounds putterines A (**244**) and B (**245**) have been separated from the roots of *M. putterlickoides* [76]. The compound 7-(acetyloxy)-*O*^11^-benzoyl-*O*^2,11^-deacetyl-7-deoxoevonine (**246**) was isolated from the methanol extracts of the barks of the Colombian medicinal plant, *M. laevis* [52]. Chiapenines ES-I (**247**), ES-II (**248**), ES-III (**249**) and ES-IV (**250**) were isolated from the leaves of *M. chiapensis* [87]. Compound jelskiine (**251**) was obtained from *M. jelskii* and *M. cuzcoina* [88]. Compounds *O*^9^-benzoyl-*O*^9^-deacetylevonine (**252**) and 8*β*-acetoxy-*O*^1^-benzoyl- *O*^1^-deacetyl-8-deoxoevonine (**253**) have been separated from the organic extracts of the roots of *M. spinosa* [24]. Compounds 1*α*,2*α*,6*β*,8*β*,9*α*,15-hexacetoxy-4*β*-hydroxy-3*β*,13-[2′-(3-carboxybutyl)] nicotinicacid-dicarbo-lactone-*β*-dihydroagarofuran (**254**), 1*α*,2*α*,9*α*,15-tetracetoxy-4*β*,6*β*-dihydroxy-8-oxo,3*β*,13-[4′-(3-carboxybutyl)]nicotinicacid-dicarbolactone-*β*-dihydroagarofuran (**255**), 1*α*,2*α*,9*α*,15-tetracetoxy-4*β*,6*β*,8*β*-trihydroxy-3*β*,13-[4′-(3-carboxybutyl)] nicotinicacid-dicarbolactone-*β*-dihydroagarofuran (**256**) and 1*α*,2*α*,8*β*,9*α*,15-pent acetoxy-4*β*,6*β*-dihydroxy-3*β*,13-[4′-(3-carboxybutyl)] nicotinicaciddicarbolactne-*β*-dihydroagarofuran (**257**) were isolated from the leaves of *M. spinosa* [65]. Compounds 4-deoxyalatamine (**258**), 1-*O*-benzoyl-1-deacetyl-4-deoxy-alatamine (**259**), 1,2-*O*-dibenzoyl-1,2-deacetyl-4-deoxyalatamine (**260**) and 4-deoxyisowilfordine (**261**) were obtained from an ethyl acetate extract of *M. oblongata* stems [31] (Table 6 and Figure 6).

#### 2.3.2. Maytansinoids

**Figure 7:**
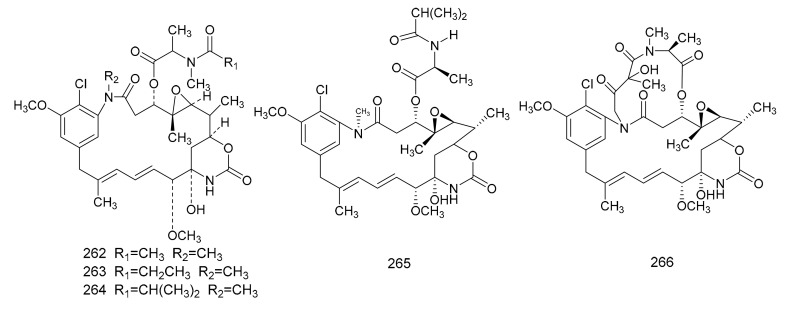
The chemical structures of *maytansinoids*, isolated from *Maytenus*.

In 1972, Kupchan et al. [7] found a macrolide alkaloid, maytansine (**262**), which was a natural product that had anti-tumor activities, and this was first isolated from *M. serrata*. Compound **262** is an anti-tumor agent with a novel structure, and, therefore, is of great clinical interest. Subsequently, maytansine (**262**), maytanprine (**263**) and maytanbutine (**264**) were isolated from *M. buchananii* [89]. Larson et al. [90] also isolated two new maytansinoid compounds, 2′-*N*-demethylmaytanbutine (**265**) and maytanbicyclinol (**266**) from *M. buchananii* (Figure 7).

## 3. Chemical Synthesis of Maytansine and Maytansine Fragments

Maytansine and its homologues are extremely rare in nature, consisting of only two ten millionths of all the constituents of *Maytenus* plants. Owing to its unique skeleton and remarkable bioactivities, maytansine has attracted many synthetic endeavors, in order to construct its core structure in the laboratory. Thus far, several synthetic studies of only the partial structure of maytansine have been reported.

**Figure 8:**
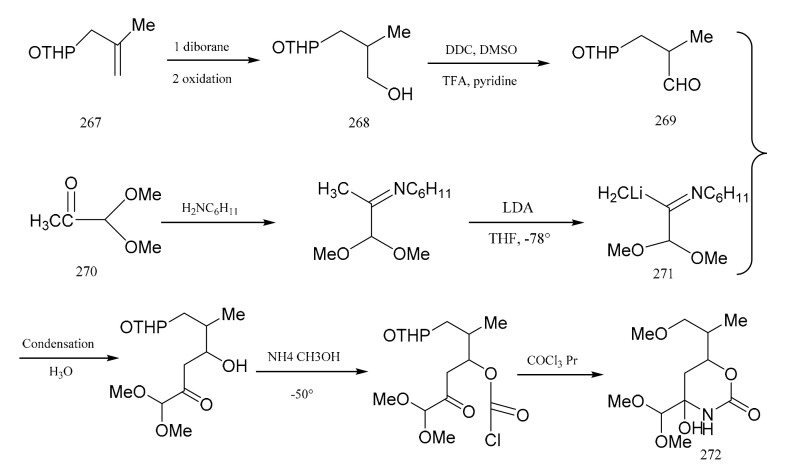
The synthetic by-products of the eastern fragments of maytansine.

Meyers and his colleagues divided maytansine into four partial structures, referring to them as the northern (**272**), eastern (**279**), southern (**307**) and western (**298**) zone fragments. Meyers et al. [91] then reported a synthetic method for the eastern fragment, the cyclic carbinolamide (**272**). Treatment of tetrahydropyranyl (**267**) with diborane gave the primary alcohol (**268**), which was then further oxidized to the aldehyde (**269**). Starting from pyruvaldehyde dimethyl acetal product (**270**), lithio-imine (**271**) was prepared through two steps. Condensation of the aldehyde (**269**) with the lithio-imine (**271**) produced the cyclic carbinolamide (**272**) (Figure 8).

**Figure 9:**
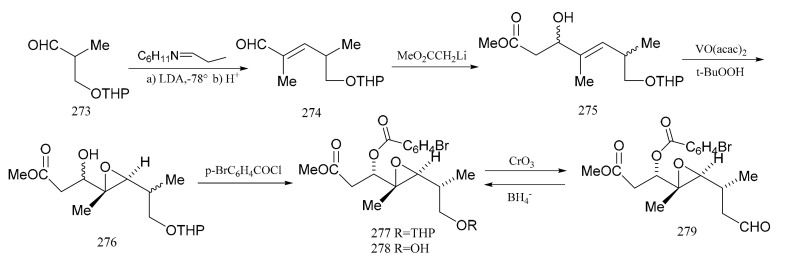
The synthetic by-products of the northern fragments of maytansine.

Meyers et al. [92] subsequently described a stereo-selective synthesis of the “northern zone” (**279**), with all its attending stereo-chemistry corresponding to the contiguous carbon chain C-1 to C-7 of maytansine. Treatment of the aldehyde (**273**) with the cyclohexylamine of proplonaldehyde followed by dehydration produced the unsaturated aldehyde (**274**). Further condensation of compound **274** with lithio methylacetate furnished the *β*-hydroxy ester (**275**) as a mixture of diastereomers. This mixture was transformed into the epoxide (**276**) using t-butylhydroperoxide, in the presence of vanadium acetylacetonate. Treatment of the epoxide (**276**) with p-bromobenzoyl chloride (ether-pyridine) produced the p-bromobenzoate (**277**), which was then hydrolyzed directly to the alcohol (**278**) as a component in a mixture of four diastereomers. Product (**278**), which accounted for 42% of the total epoxide mixture, was the major component of the isomeric mixture obtained. Oxidation of these products gave a single aldehyde (**279**), and this stereo-selective synthesis of compound **279** provided an ample supply of the “northern zone” fragment for further studies (Figure 9).

**Figure 10:**
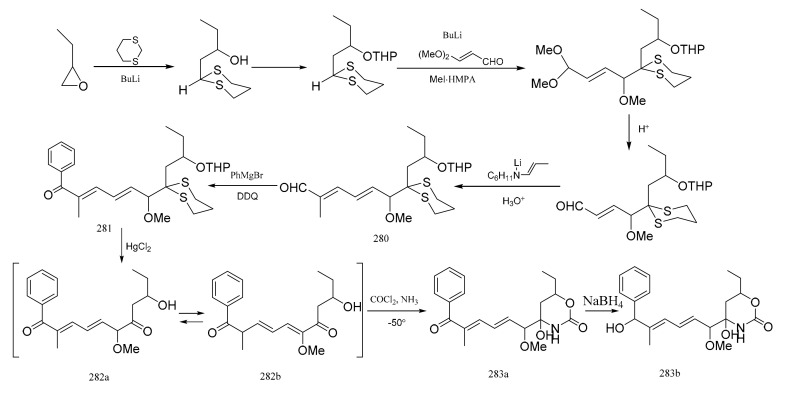
Some alternative synthetic by-products of the northern fragments of maytansine using two Wittig aldol condensation reactions.

Meyers et al. [93] was able to synthesize the corresponding products to the C7–C16 fragment of maytansine. E,E-dienal (**280**) was prepared via two Wittig aldol condensations. After treatment of compound **280** with phenylmagnesium bromide, the mixture was oxidized without further purification to the ketone (**281**). Removal of the dithiane and tetrahydropyranyl protective groups in (**281**) was accomplished in a single step using an acetonitrile water mixture, which led to the production of compound **282** as an equilibrated mixture of two similar compounds (**282a**:**282b**=2:8). The mixture was then treated with phosgene, and then with methanolic ammonia which produced the cyclic carbamate (**283a**). Reduction of compound **283a** with sodium borohydride to compound **283b** corresponded to the exact southern portion of colubrinol, an ansa-macrolide, which differed from the maytansines only at the C-15 position (Figure 10).

**Figure 11:**
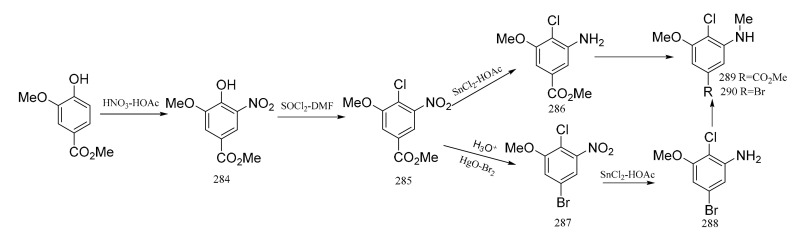
The synthetic by-products of the southern fragments of maytansine.

Meyers et al. [94] also synthesized the “western” zone products of maytansine, which contains an unusual aromatic substitution array. Methyl vanillate was used as the raw material, and it was nitrated to give the nitro derivative (**284**). Treatment of compound **284** with a thionyl chloride–dimethylformamide complex produced the chloro derivative (**285**), and reduction of this gave the aniline product (**286**). Monomethylation of compound **286** produced the *N*-methyl derivative (**289**), which could be coupled to the “southern” zone fragment through an organometallic reaction. On the other hand, hydrolysis of compound **284** and then treatment with a mercury oxide-bromine mixture gave the bromide (**287**). Reduction of compound **287** gave the aniline (**288**), which could be monomethylated, as above, to compound **290**. The acquisition of compound **290** can be utilized in coupling to the “southern” zone of maytansine via its organolithium or a Grignard derivative (Figure 11).

**Figure 12:**
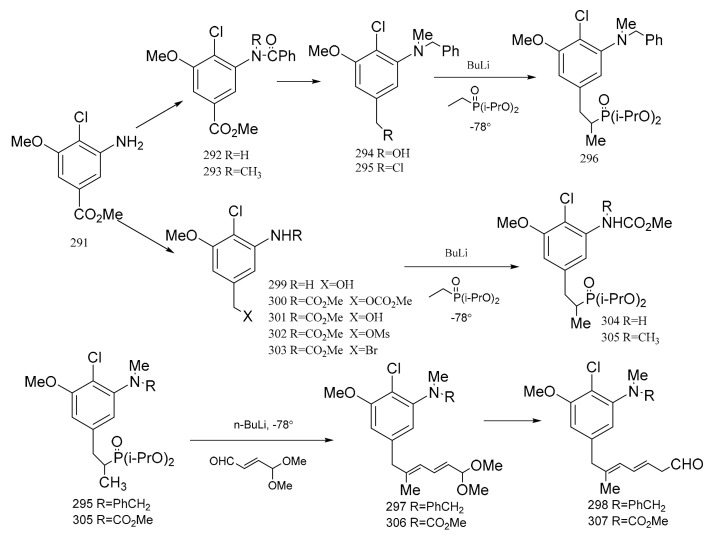
The synthetic by-products of the western-southern fragments of maytansine.

Meyers et al. [95] prepared two major precursors, (**298**) and (**307**) (corresponding to the western-southern zone of maytansine), according to the reactions shown in Figure 10. The aromatic compound (**291**) served as a common precursor to compounds **289** and **298** by the routes below. Treatment of the amino ester product (**291**) with benzoyl chloride triethylamine gave the *N*-benzoyl derivative (**292**). Methylation of compound **292** produced **293**, and this was then reduced with lithium aluminium hydride to give the *N*-benzyl alcohol derivative (**294**), which could be transformed into the chloride (**295**) using mesyl chloride, lithium chloride and dimethylformamide. Treatment of compound **295** with a solution of lithiated ethyl di-isopropyl phosphonate afforded the phosphonated product. Addition of an organolithium reagent, *n*-b*utyllithium,* gave compound **296** which could then be treated with E-*γ*,*γ*-dimethoxycrotonaldehyde to give the diene (**297**). This product immediately hydrolyzed to the dieneal (**298**). Reduction of compound **291** gave **299**, which was converted to the carbamate–carbonate (**300**) with methyl chloroformate. The selective removal of the carbonate gave the alcohol by-product (**301**). Compound **301** could then be transformed into the mesylate (**302**). Treatment of the mesylate (**302**) with lithium bromide in dimethylformamide gave the benzyl bromide (**303**), which was alkylated with lithio-ethyl di-isopropyl phosphonate to furnish compound **304**. Methylation of compound **304** gave the phosphonated product (**305**). The addition of *n*-b*utyllithium* to compound **305** gave the lithiated phosphonate, which could then be treated with E-*γ*,*γ*-dimethoxycrotonaldehyde to give the diene (**306**), which was immediately hydrolyzed to the dieneal (**307**) in a similar way to compounds **297** and **298**, as mentioned above (Figure 12).

**Figure 13:**
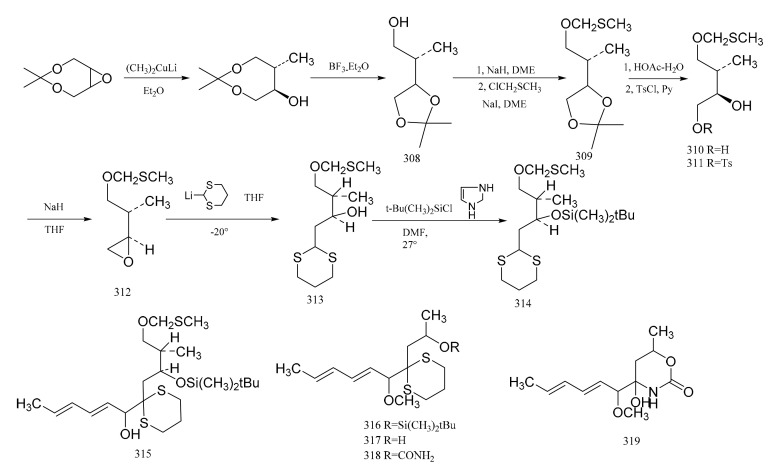
The production of compound **314** and its derivatives.

Corey et al. [96] first prepared the acyclic intermediate (**314**), which corresponds to carbons five to nine of maytansine. This intermediate (**314**) appears to be especially useful because carbons six and seven are directly associated with the generation of the stereocenters of the other compounds in this series. Using the selectivity of dimethyl copper lithium to the *trans* ring opening of the epoxide, the relative configuration of the two carbon atoms is consistent with those of carbons six to seven of maytansine. The synthetic steps are shown in Figure 13.

The *cis*-2-buten-1,4 diol was reacted in order to produce the ketal derivative (**308**), and then the hydroxyl group of compound **308** was protected by forming the methylthiomethyl ether (**309**). Exposure of compound **309** to a mixture of acetic acid and water gave the diol (**310**), which could be converted via the monotosylate (**311**) to the epoxide (**312**). Reaction of (**312**) with 2-lithio-1,3-ditbiane produced the hydroxy dithiane (**313**), which was further transformed into the silyl ether derivative (**314**) by reaction with t-butyldimethylsilychloride-imidazole in dimethylformamide. Treatment of compound **314** with n-butyllithium and tetra-methylethylenediamine led to a lithio derivative; then, the addition of sorbaldehyde gave the dienol (**315**), which was transformed into the corresponding methyl ether by treatment with sodium hydride and methyl iodide. Taking advantage of the unique susceptibility of the methylthiomethyl ether protecting group to fusion, the removal of this protective group to form the corresponding alcohol could be affected, and the silyl ether grouping was unaffected. This showed that the selective removal of the t-butyldimethylsilyl group from the oxygen by fluoride ion occurred through the model system (**316**), and the 1,3-dithiane was unaffected. Compound **316** was therefore converted to the alcohol (**317**). Reaction of compound **317** sequentially with sodium hydride, phosgene and ammonia led to the urethane derivative (**318**). Reaction of the latter removed the 1,3-dithiane, which gave rise to the heterocycle (**319**), a compound which possesses the characteristic structure of the C(8) to C(14) section of the maytansine molecule (Figure 13).

**Figure 14:**
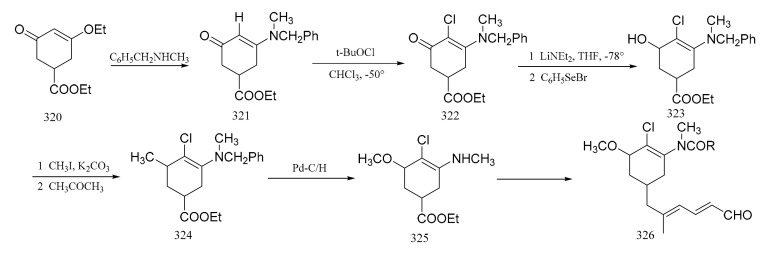
Synthesis of the benzenoid-associated intermediates of maytansine.

Corey et al. [97] reported a synthetic route of an intermediate, which corresponds to the benzenoid part of maytansine. The enone ester (**320**), which was obtained using gallic acid, was treated with *N*-methylbenzylamine to give the enamino ketone (**321**). Reaction of this ketone (**321**) with tert-butyl hypochlorite in chloroform formed the 2-chloro derivative (**322**). The benzoic ester (**323**) was then obtained by aromatization of compound **322** with lithium diethylamide and benzeneselenyl bromide in tetrahydrofuran. Treatment of compound **323** with methyl iodide and potassium carbonate in acetone gave the phenolic methyl ether (**324**), which subsequently underwent hydrogenolysis quantitatively to give the desired amino ester (**325**). This was followed by the further elaboration of compound **325** to the dienal (**326**) (Figure 14).

**Figure 15:**
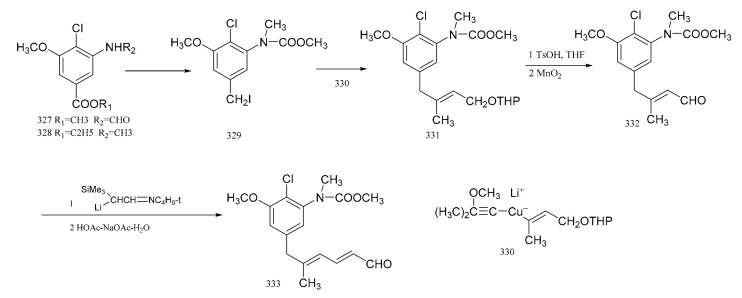
Synthesis of strategic intermediates corresponding to the C10-N fragment of maytansine.

**Figure 16:**
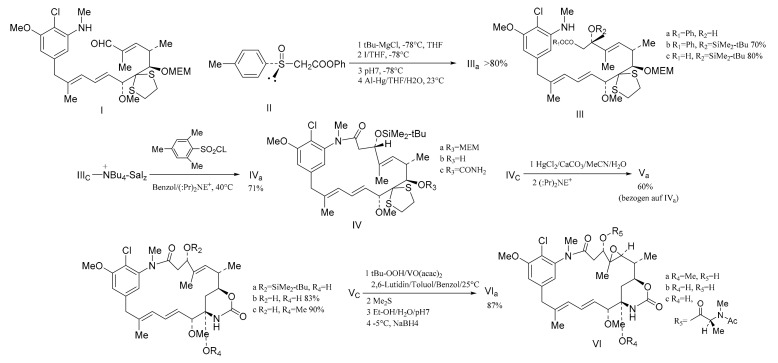
The stereo-selective synthesis of maytansine.

Corey et al. [98] used a high degree of stereo-selectivity in order to synthesize a strategic intermediate corresponding to C10-N fragment of maytansine. Starting from the raw materials, either (**327**) or (**328**), the iodide (**329**) was prepared through three steps. The iodide (**329**) was transformed efficiently and selectively into the E-tri-substituted olefinic derivative (**330**), through a cross coupling reaction with a specially designed mixed Gilman reagent (**331**) (which involved the use of a cuprate). The mixed Gilman reagent (**331**) was highly soluble, which allowed the whole process to be conducted under homogeneous conditions in a tetrahydrofuran solution. This led to a higher yield of coupling product. Oxidation of compound **330** produced the aldehyde (**332**). The *α*-trimethylsilyl derivative of acetaldehyde *N*-*t*-butylimine was converted to the *α*-lithio derivative, by reaction with sec-butyllithium in dry ether under argon. This allowed the aldehyde (**332**) to react and form the dienal derivative (**333**) with an 80% yield (Figure 15). Corey’s group attempted to synthesize molecular fragments of maytansine in 1972. Finally, in 1980, the group was able to stereo-selectively synthesize maytansine [99] (Figure 16).

**Figure 17:**
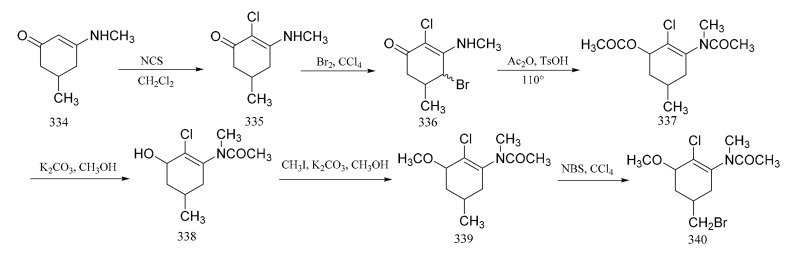
Synthesis of the aromatic portion of maytansine.

Foy and Ganem [100] reported on the synthesis of the aromatic portion of maytansine via a six-step route. Condensation of 5-methylcyclohexane-1,3-dione with aqueous methylamine formed compound **334**, which could be chlorinated with N-chlorosuccinylmide in dichloromethane to result in a chloroenaminoketone derivative (**335**). This could be further oxidized by the addition of bromine in carbon tetrachloride. A mixture of monochlorobromides (**336**) was formed during this reaction, from which one isomer then crystallized. The mixture could then be treated directly with a mixture of acetic anhydride and p-toluenesulfonic *acid*, to affect its dehydrobromination. Saponification of compound **337** gave the corresponding phenol (**338**), which was methylated to give the methoxyacetanilide (**339**). Oxidation of compound **339** and subsequent reaction with *N-bromosuccinimide* produced compound **340** (Figure 17).

**Figure 18:**
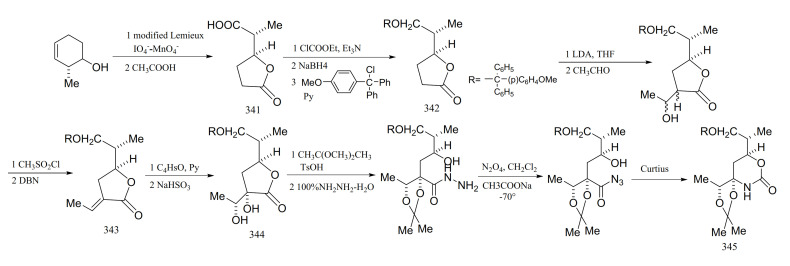
Compounds synthesized from the cyclic carbamate unit of maytansine.

Edwards and Ho [101] reported a relatively efficient new approach to the synthesis of the derivatives from the cyclic carbamate unit in maytansine, which involved the successful introduction of the required four asymmetric centers with complete stereo-specificity. The starting material in the synthesis was 3,4-epoxycyclohexene, and the cyclic carbamate was prepared via a series of eleven steps. Through selective reduction of the *γ*-lactone derivative (**341**) and protection of compound **342** to form an intermediate (**343**) (mainly composed of formula *E*), the diol compound formed (**344**) was protected, and a Curtius re-arrangement of the azide gave the cyclic carbamate (**345**) (Figure 18).

**Figure 19:**
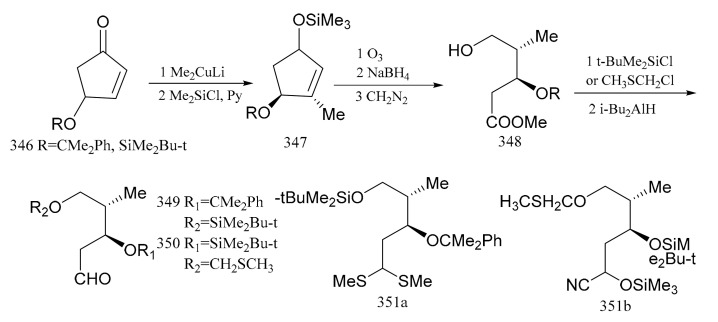
Compounds synthesized from the (*S*)-(+)-4-hydroxy-2-cyclopentenone derivative of maytansine.

Samson et al. [102] used (*S*)-(+)-4-hydroxy-2-cyclopentenone, which was readily available from the reaction with (*R,R*)-(+)-tartaric acid, as the starting material in the synthesis which was converted via the intermediates (**346**, **347** and **348**) to the aldehydes (**349** and **350**) with the same configuration as C6 and C7, respectively. When the aldehyde group in compound **349** was treated with trimethylorthothioborate, compound **351a** was produced. The aldehyde group in compound **350** could be treated with trimethylsilylcyanide to yield the protected product, cyanohydrin (**351b**), as a mixture of two diastereoisomers. Both compounds, **351a** and **351b,** can be regarded as potential acylanion equivalents (Figure 19).

**Figure 20:**
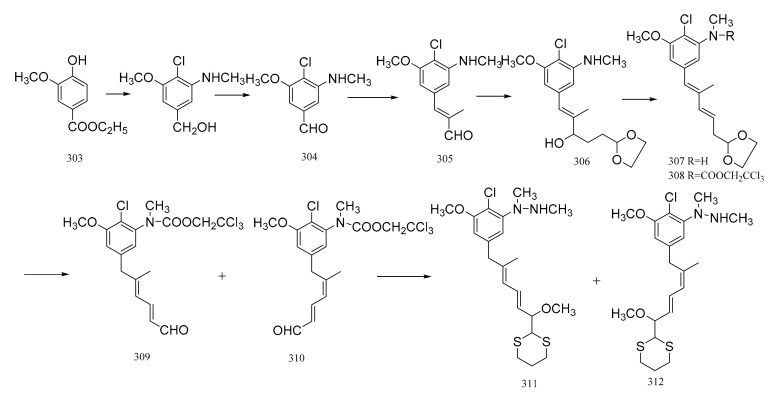
Synthesis of the substituted aromatic portion and the C9 to C15 moiety of maytansine.

Gotschi et al. [103] have synthesized the correctly substituted aromatic portion of maytansine, as well as its C9 to C15 moiety. The synthesis was initiated from the ethyl vanillate derivative (**352**) via the aldehyde (**353**). The aldehyde (**353**) was then condensed with propionaldehyde to (**354**). The elongation of the sidechain was realized by the reaction of compound **354** with Grignard reagent yielding the alcohol (**355**), which was dehydrated to form the diene (**356**). The hydrolysis of the acetal function necessitated prior acylation of the amino group. The reaction of compound **356** with 2,2,2-trichloroethoxycarbonyl chloride in pyridine, then the hydrolysis of the crude carbamate derivative formed (**357**) with 1 N aqueous hydrochloric acid/acetone, gave a 2:1 mixture of the two stereo-isomeric dienals (**358**) and (**359**). Reaction of this mixture with 2-lithio-1,3-dithiane, followed by the removal of the trichloroethoxycarbonyl group, resulted in obtaining a corresponding mixture of the stereo-isomeric of C9-N fragments of maytansine (**360** and **361**). The *E*,*E*-configuration was the major component (**360**) of the mixture of compounds formed (Figure 20).

**Figure 21:**
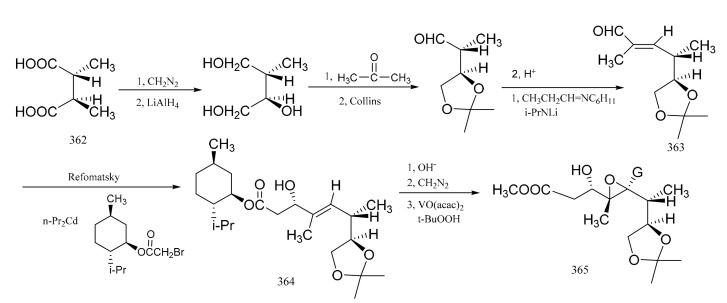
Stereo-selective synthesis of the derivatives from the C1–C8 fragment of maytansine.

Pan et al. [104] reported the stereo-selective synthesis of the C1–C8 fragment of maytansine. Starting from (-)-(*2R,3R*)-2-hydroxy-3methyl-succinic acid (**362**) as a raw material which was obtained by resolution of threo-(±)-methyl-malio acid with the aid of cinohonino, E-*α*,*β*-unsaturated aldehyde (**363**) was prepared via a multi-step reaction sequence. Reformatsky reaction of the aldehyde derivative (**363**) with (-)-menthyl bromoacetate in the presence of n-propyl cadmium formed the *β*-hydroxyester, which was then hydrolyzed to the free acid and then re-esterified with diazomethane to give the b-hydroxy methyl ester (**364**). Compound **364** was mainly of the S configuration. Acetylation of compound **364** with acetic anhydride in the presence of pyridine followed by epoxidation of the double bond with vanadium acetyl acetonate and *t*-butyl hydrogen peroxide gave compound **365**, which had the required configuration. The C1–C8 fragment had five ohiral centers in the natural configuration (Figure 21).

**Figure 22:**
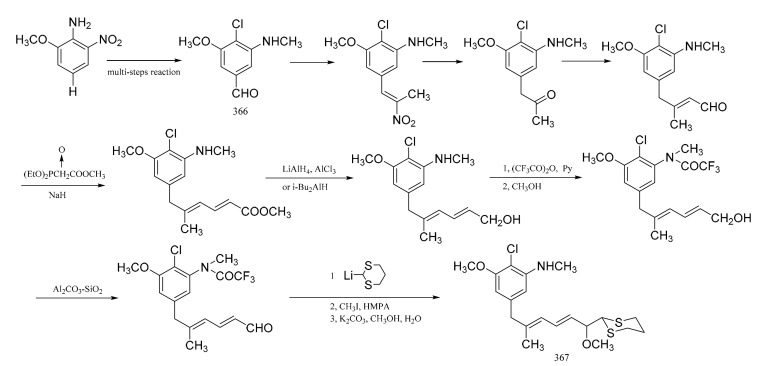
Synthesis of the derivatives from the C9-N fragment of maytansine.

Zhou et al. [105] were able to synthesize the corresponding derivatives to the C9-N fragment of maytansine. Starting from 2-methoxy-6-nitroaniline, 4-chioro-3-methoxy-5-methyl-aminobenzaldehyde (**366**) was prepared through six separate steps. The long side chain in (**367**), with high stereo-specificity, was derived from the aldehyde group in compound **366**, via a reaction sequence of twelve steps (Figure 22).

**Figure 23:**
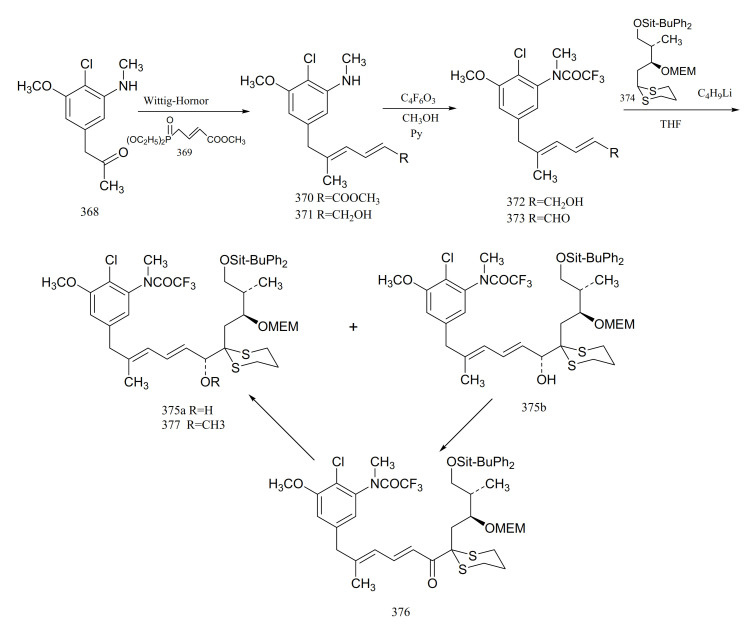
Synthesis of the derivatives from the C5-N fragment of maytansine.

Gu et al. [106] reported the synthesis of an important intermediate, the C5-N fragment of maytansine. This fragment possesses an aromatic moiety, two conjugated *trans* double bonds and three asymmetrical carbons, as well as the C6, C7 and C10 of maytansine. A Wittig–Horner reaction of compound **368** with **369** gave the aromatic diene ester derivative (**370**). Reduction of compound **370** to the dienol (**371**) was followed by treatment with trifluoroacetic anhydride and methanol, to give the trifluoroacetamide (**372**). Oxidation of compound **372** formed the dienal (**373**). Lithiation of compound **374** with n-butyllithium, which could then be coupled with compound **373** to afford a pair of epimers (**375a** and **375b**). Oxidation of (**375b**) with active manganese dioxide in methylene chloride produced the corresponding ketone (**376**). Reduction of compound **376** with the R-binaphthol-lithium aluminum hydrogen-ethanol complex produced compound **375a**, which could be methylated with sodium hydride and methyl iodide in tetrahydrofuran to give the C5-N fragment (**377**) of maytansine (Figure 23).

**Figure 24:**
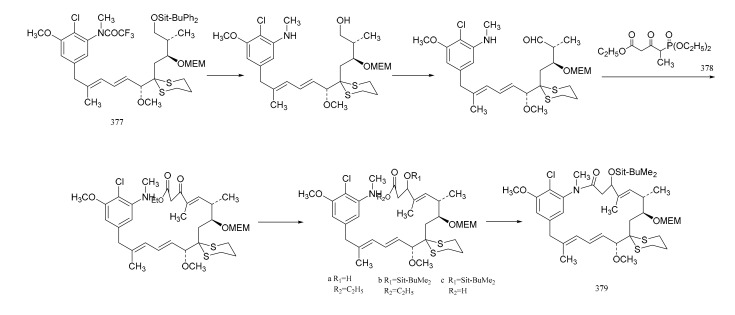

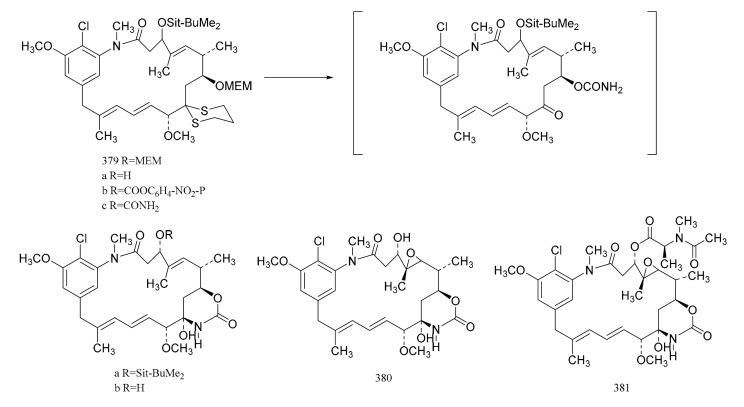
The final steps in the synthesis of maytansine.

Gu et al. [107] also linked the C5-N fragment (**377**) with compound **378**, and a ring closure led to the production of the macrolide (**379**); then, the selective removal of the protective group formed the small ring lactone and epoxidation of the product gave the maytansinol (**380**). Finally, the introduction of side chain amino acids led to the synthesis of maytansine (**381**) (Figure 24).

## 4. Pharmacological Activities

### 4.1. Antitumor Activities

Scholars at home and abroad have successively isolated the maytansinoids from the genus *Maytenus* since the 1970s. These compounds have high anti-tumor activities, and their properties were proven through clinical trials and subsequent application for a number of years. The Chinese Cancer Institute has conducted medical studies [8] to prove that the effective constituents isolated from *M. hookeri* Loes have strong anti-cancer activity, and they published that these compounds are able to inhibit cancer cells undergoing the final disintegration in the middle stage of cell mitosis. Clinical trials have shown that crude methanol extracts of *M. hookeri* Loes have significant effects on lympho-sarcomas, peritoneal mesotheliomas as well as multiple myelomas. Other studies outside China have shown that maytansine is active against human nasopharyngeal carcinoma cells, melanoma B16 cells and mouse leukemia L1210. It has also been shown to have effects on P388 tumors, mast cell tumor P815, plasmacytoma YPC-1 and rat W-256 carcinosarcoma. In the first clinical trial, maytansine was shown to have anti-tumor activity against lymphocytic leukemia, lymphoma, ovarian cancer, breast cancer and melanoma [9]. Hiroshi et al. [10] isolated triterpenes from the bark of *M. chuchuhuasca*, all of which were shown to markedly inhibit the polymerization of tubulin. Kuo et al. [11] reported that emarginatine F demonstrated strong cytotoxicity against human epidermoid carcinoma of the nasopharynx (KB), ileocecal adenocarcinoma (HCT-8), melanoma (RPMI-7951) and medulloblastoma (TE-671) tumor cells, as well as against murine leukemia (P-388).

The researchers at the 62nd Hospital of the People’s Liberation Army [108] used tablets and decoction of *M. hookeri* Loes methanol extracts for clinical treatment, and conducted the necessary animal tests. Clinical treatment was also performed on 17 cases of malignant tumors in 14 different cancers, including leukemia, lymphocytic cell tumors, nasopharyngeal carcinoma, lung cancer, esophageal adenocarcinoma and liver cancer. The results of animal experiments showed it inhibited ascites-type liver cancer and rat Wacker carcinoma in mice. The clinical studies showed that the methanol extracts and decoction resulted in varying degrees of efficacy in 10 cases of malignant tumors of the 14 diseases, including tumor shrinkage, symptom reduction and increased appetite. Among the patients, two cases were markedly effective and eight cases were described as effective. It is believed that the constituents of *M. hookeri* Loes are a promising anti-cancer botanical drug. Ning et al. [109] studied the changes of morphology and structure of the epithelial cell line, Eca109, which was derived from a type of human esophageal cancer. They found that the effects of maytansine treatment on cancer cells were similar to those caused by microtubule inhibitors, such as vincristine. This suggests that maytansine is an alternative microtubule inhibitor with anti-tumor effects.

Fan et al. [110] showed that the ethyl acetate extract 761-1 of *M. confertiflorus* had an effect on transplanted animal tumors, such as EAC, L7212 and W256, and the spermatogonium are positive. The stem portion M2 was also effective for tumors, such as EAC, HepA and W256. The anti-cancer compound, maytansine, was found to be effective for EAC, HepA, L1210, S180, B16 melanoma and W256. Gong et al. [111] showed that extracts of *M. hainanensis* can induce and differentiate tumor cells in the human body, leading to an effective inhibition of the synthesis of tumor cell DNA. Nabende et al. [112] showed that the extracts of *M. senegalensis* showed a certain degree of anti-proliferative activity in breast cancer and colon cancer cells, although they were not toxic to Vero cells. Zeng et al. [113] reported that the compounds isolated from *Maytenus* had significant anti-cancer activities against human cancer H226 and HeLa cells, both in vitro and in vivo, highlighting that they may be an anti-cancer medicine. Compound (16*β*)-16-hydroxy-pristimerin was isolated from *M. salicifolia*, which exhibited an anti-proliferative effect on HeLa, A-549 and HL60 human cell lines [29]. Maytenfoliol was separated from *M. diversifolium*, which showed significant anti-leukemic activity [37]. Chavez et al. [61] isolated 6*β*,8*β*-15-triacetoxy-1*α*,9*α*-dibenzoyloxy-4*β*-hydroxy-*β*-dihydroagarofuran and 1*α*,6*β*,8*β*,15-tetraacetoxy-9*α*-benzoyloxy-4*β*-hydroxy-*β*-dihydroagarofuran from the aerial parts of *M. macrocarpa*, and both compounds showed marginal anti-tumor activities against four cell lines grown in cell culture.

### 4.2. Anti-Bacterial Activities

Gonzalez et al. [12] isolated 6-oxo-iguesterol, 6-oxo-tingenol and 3-*O*-methoxy-6-oxo-tingenol from the root barks of *M. canariensis*. Three compounds showed antibiotic activities against *B. subtilis*, with minimal inhibitory concentrations (MICs) of 12–14, 35–39 and 25 μg/mL, respectively. 6-oxo-tingenol was also active against *Staphylococcus aureus* with a MIC of 40–50 μg/mL. Alvarenga et al. [17] obtained a new nortriterpene quinone methide, 15α-hydroxy-21-keto-pristimerine, from the root barks of *M. catingarum*, which showed potent activity against Gram-positive bacteria. A new norquinonemethide triterpene with a netzahualcoyene type skeleton, scutione, was isolated from the root barks of *M. scutioides*, which showed antibiotic activity against Gram-positive bacteria [34]. Muhammad et al. [51] isolated the oleanane triterpenoid, koetjapic acid, from *M. undata*, which inhibited the growth of *S. aureus*, including a penicillin-resistant strain of this bacteria as well as *Pseudomonas aeruginosa*, with a MIC range of 3.125–6.25μg/mL. Ni et al. [114] isolated the fungal strain, *Chaetomium globosum* Ly50′, from the leaf of *M. hookeri* Loes, which showed anti-bacterial activity. The fermentation extracts of this strain yielded two compounds that were active against *Penicilium avellaneum*, UC-4376 and *Mycobacterium tuberculosis*, and these were determined to be chaetoglobosins A and B, respectively. Wu et al. [115] demonstrated that maytansine actively inhibited the growth of eukaryotic cells, and had anti-fungal activities against some pathogenic plant fungi. Maytansine can be absorbed by the leaves of Chinese cabbage, and it has been also shown to have anti-bacterial effects, while the latter also had anti-microbial activity [116]. Liu et al. [117] isolated a lignan tanegool from the 95% ethanol extracts of *Gymnosporia varialilis* that also had anti-bacterial activity. The results showed that the inhibition ratio against *Selerotinia scleotiorum*, *Bipolaris sorokiniana*, *Alternaria solani* and *Fusarium oxysporum* f. sp. *niveum* were more than 75%, when the concentration of *G. varialilis* Loes used was 10 mg/mL.

### 4.3. Other Pharmacological Activities

A new compound 3-*O*-methyl-6-oxo-pristimerol was isolated from the hexane/Et_2_O 1:1 extracts of the root barks of *M. chubutensis*, which showed moderate multidrug-resistance reversal activity [26]. Zhang et al. [118] found that the 95% ethanol extracts of the aerial parts of *G. varialilis* Loes showed angiotensin-converting enzyme (ACE) inhibitory activity, and this could be allocated to two active compounds from *G. varialilis* Loes. These were (+)-catechin and caffeic acid. Hamisi et al. [119] found that the ethanolic extracts of root barks of *M. senegalensis* possessed potent anti-plasmodial effects, and may, therefore, serve as a potential source of an alternative safe, effective and affordable anti-malarial drug. Bishnoi [120] studied the anti-hyperglycemic activity of the hydroalcoholic extracts of the leaves of *M. emarginatus*. The results showed that the dried extracts *M. emarginatus* (250 and 500 mg/kg) significantly reduced the levels of blood glucose comparable to glibenclamide (10 mg/kg), a well-known glyburide, which is a medication used to treat type 2 diabetes mellitus. Thus, Bishnoi and his colleagues concluded that the extracts of the leaves of *M. emarginatus* had anti-hyperglycemic activity. In addition, the plants of *Maytenus* have long been used as a traditional Chinese medicine for the treatment of anti- inflammatory [51] conditions, as well as HIV infections [121]. 1*α*,2*α*,9*β*,15-tetracetoxy-8*β*-benzoyloxy-*β*-dihydroagarofuran was isolated from the leaves of *M. spinosa*, and it was shown to have anti-HIV activity [65]. Elmer et al. [5] reported that *M. macrocarpa* leaves had anti-inflammatory activity and concomitant neuro-behavioral side-effects. Joshi et al. [122] reported that the plant, *M. emarginata,* could be used as a potential antioxidant.

## 5. Toxic Effects

The American Cancer Research Institute [9] found that the main toxicity of maytansine was against the gastrointestinal system and included nausea, vomiting and diarrhea. This was discovered in phase I~II clinical trials, and the effects were directly related to the dose administered. Hepatotoxicity was manifested as a clinically insignificant transient rise of some liver enzymes with the appearance of jaundice. Neurotoxicity was experienced at both the central and peripheral levels, and toxicity of the central nervous system was characterized by dizziness, anxiety and insomnia, together with peripheral neurotoxic symptoms, such as paresthesia as well as muscle pain and weakness. Hematological toxicity was found to be uncommon, and usually manifested as transient thrombocytopenia and myelosuppression; however, symptoms were slight, reversible and unrelated to dose used. The studies from the 62nd Hospital of the People’s Liberation Army [108] involved only a few people who took the methanol extracts of *M. hookeri* Loes. Afterwards, they generally experienced a slightly higher level of thirst, when even a single decoction was taken on an empty stomach. Also, a few people had some light nausea. Meneguetti et al. [123] found that *M. guyanensis* did not present genotoxic effects during their animal experiments.

## 6. Conclusions

This paper systematically summarizes the advances made in the research on the medicinal effects of *Maytenus*, with respect to its chemical constituents and pharmacological activities. Its structural diversity is linked to its biological diversity. The carbonyl group in the E ring of friedelane triterpenoids and the presence of the hydroxyl and C(21)=O groups enhances its anti-bacterial activity. The C-28 carboxyl of triterpenes is usually an important group for conferring its cytotoxic activity. The antibiotic activity of friedelane triterpenoids may be associated with the presence of free hydroxyl groups in the ring A. For sesquiterpenes and its alkaloids with the same skeletons, its biochemical activity was found to vary with the nature of the esterification residues present. The greater the number of acetyl residues, the greater the anti-feedant activity. The opposite was observed with the benzoyl residues, where increasing the frequency of these groups was observed to decrease anti-feedant activity. Insecticidal activity was roughly correlated with the presence of a carbonyl group at the C-8 position. Cytotoxicity was noticeably affected by the type of functional group substitution in sesquiterpene pyridine alkaloids at the C-1 and C-9 positions, and by the configuration of the proton at position C-8 (cz or fi). The furoyloxy groups in the dihydro-β-agarofuran sesquiterpenoids at positions C-6 and C-9 seemed to be important in the modulation of NF-κB, which correlates with the anti-inflammatory activity. Based on the accumulated information and the advancements in synthetic methods, maytansine can be increasingly important as an anti-cancer drug. Any adverse reactions can potentially be reduced through structural modifications of the molecule. This compound is expected to give rise to new anti-tumor drugs, and these will be at the forefront of our endeavors to combat cancer. Due to the existence of the rich active ingredients present in *Maytanus*, researchers have not stopped exploring and researching the many and varied bioactivities of the compounds from this valuable plant resource.

## Figures and Tables

**Table 1:** The friedelane triterpenes isolated from *Maytenus*.

No.	Name	R_1_	R_2_	R_3_	R_4_	R_5_	R_6_	R_7_	R_8_	Type	Ref.
**1**	Pristimerin	H	H	COOMe	H	H	-	-	-	A1	[16]
**2**	15*α*-hydroxy-21-keto-pristimerine	*α-*OH	H	COOMe	=O	H	-	-	-	A1	[17]
**3**	2,3,22*β*-trihydroxy-24,29-dinor-1,3,5(10),7-friedelatetraene-6,21-dione-23-al	OH	OH	CHO	H	=O	*β-*OH	-	-	A2	[18]
**4**	2,22*β*-dihydroxyl-3-methoxy-24,29-dinor-1,3,5(10),7-friedelatetraene-6,21-dione	OH	OH	CH_3_	H	=O	*β-*OH	-	-	A2	[18]
**5**	2,3,22*β*-triihydroxy-23,24,29-trinor-1,3,5(10),7-friedelatetr aene-6,21-dione	OH	OH	H	H	=O	*β-*OH	-	-	A2	[18]
**6**	2,22*β*-dihydroxyl-3-methoxy-24,29-dinor-1,3,5(10),7-friedelatetraene-6,21-dione	OH	OCH_3_	CH_3_	H	=O	*β-*OH	-	-	A2	[18]
**7**	2,3,22*β*-trihydroxy-24,29-dinor-1,3,5(10)–friedelatetraene -6,21-dione	OH	CH_3_	=O	H	H	H	=O	*β-*OH	A3	[18]
**8**	2,15*α*,22*β*-trihydroxy-3-methoxy-24,29-dinor-1,3, 5(10)-friedelatriene-21-one	OCH_3_	CH_3_	H	H	*α*-OH	H	=O	*β-*OH	A3	[18]
**9**	3,22*β*-dihydroxy-24,29-dinor-l(10)-3,5-friedelatriene-2,7,21-trione	=O	*β-*OH	=O	H	-	-	-	-	A4	[18]
**10**	3,22*β*-dihydroxy-24,29-dinor-l(10),3,5–friedelatriene-21-one	H	*β-*OH	=O	H	-	-	-	-	A4	[18]
**11**	2,3,22*β*-trihydroxy-24,29-dinor-25(9→8)-1,3,5(10),7-friedelatetraene-21-one-23-al	*β-*OH	-	-	-	-	-	-	-	A5	[18]
**12**	23-oxo-iso-tingenone	H	-	-	-	-	-	-	-	A5	[19]
**13**	(8*S*)-7,8-dihydro-7-oxo-tingenoe	=O	H	=O	H	-	-	-	-	A4	[19]
**14**	(7*S*, 8*S*)-7-hydroxy-7,8-dihydro-tingenone	*α*-OH	H	=O	H	-	-	-	-	A4	[19]
**15**	(8*S*)-7,8-dihydro-6-oxo-tingenol	OH	CH_3_	=O	H	H	H	=O	H	A3	[19]
**16**	23-nor-6-oxo-tingenol	OH	OH	H	H	=O	H	-	-	A2	[19]
**17**	28-hydroxy-friedelane-1,3-dione	=O	H	H	H	CH_2_OH	CH_3_	CH_3_	-	A6	[20]
**18**	Macrocarpin A	OH	CHO	=O	H	H	COOMe	H	H	A3	[21]
**19**	Macrocarpin B	OH	OH	COOH	H	=O	*β-*OH	-	-	A2	[21]
**20**	Macrocarpin C	OH	OCH3	COOH	H	=O	*β-*OH	-	-	A2	[21]
**21**	Macrocarpin D	*α*-OH	*β*-OH	=O	H	-	-	-	-	A4	[21]
**22**	Maytenfolone	-	-	-	-	-	-	-	-	A7	[22]
**23**	6-oxo-iguesterol	-	-	-	-	-	-	-	-	A8	[12]
**24**	6-oxo-tingenol	OH	OH	CH_3_	H	=O	H	-	-	A2	[12]
**25**	3-*O*-methoxy-6-oxo-tingenol	OH	OCH_3_	CH_3_	H	=O	H	-	-	A2	[12]
**26**	Blepharotriol	OH	OH	OH	COOMe	H	H	-	-	A2	[23]
**27**	6-deoxoblepharodol	OH	CH_3_	H	H	H	COOMe	H	H	A3	[23]
**28**	Isoblepharodol	OH	CH_3_	H	=O	H	COOMe	H	H	A3	[23]
**29**	7-oxo-blepharodol	OH	CH_3_	=O	=O	H	COOMe	H	H	A3	[23]
**30**	15*α*-hydroxy-tingenone	*α*-OH	H	H	=O	H	-	-	-	A1	[24]
**31**	15-dihydro-pristimerin	-	-	-	-	-	-	-	-	A9	[24]
**32**	Vitideasin	*α*-COOCH_3_	H	-	-	-	-	-	-	A10	[24]
**33**	20*β*-hydroxy-scutione	*α*-OH	=O	-	-	-	-	-	-	A10	[24]
**34**	7-oxo-7,8-dihydro-scutione	-	-	-	-	-	-	-	-	A11	[25]
**35**	6,23-dioxo-7,8-dihydro-pristimerol-23-oic Acid	OH	COOH	=O	H	H	COOMe	H	H	A3	[25]
**36**	23-nor-blepharodol	OH	H	=O	H	H	COOMe	H	H	A3	[25]
**37**	3-methoxy-6-oxo-tingenol-23-oic Acid	OH	OCH_3_	COOH	H	=O	H	-	-	A2	[25]
**38**	Retusonine	-	-	-	-	-	-	-	-	A12	[25]
**39**	21-Oxopristimerine	H	H	COOMe	=O	H	-	-	-	A1	[25]
**40**	3-*O*-methyl-6-oxo-pristimerol	OH	OCH_3_	CH_3_	COOMe	H	H	-	-	A2	[26]
**41**	3*β*,24-epoxy-2*α*,3*α*-dihydroxy-D:A-friedooleanan-29-oic acid methyl ester	OH	OH	H	-	-	-	-	-	A13	[27]
**42**	2*α*-acetoxy-3*β*,24-epoxy-3*α*-hydroxy- D:A-friedooleanan-29-oic acid methyl ester	OAc	OH	H	-	-	-	-	-	A13	[27]
**43**	3*α*-hydroxy- D:A-friedooleanan-28-oic acid	*α*-OH	H	H	COOH	H	-	-	-	A14	[27]
**44**	3-oxo-D:A-friedooleanan-28,30-olide	-	-	-	-	-	-	-	-	A15	[27]
**45**	3*β*,11*β*-dihydroxyfriedelane	*β*-OH	*β*-OH	H	CH_3_	H	-	-	-	A14	[28]
**46**	3,4-seco-friedelan-3,11*β*-olide	-	-	*-*	-	-	-	-	-	A16	[28]
**47**	(16*β*)-16-hydroxy-pristimerin	H	*β*-OH	COOMe	H	H	-	-	-	A1	[29]
**48**	12,16-dihydroxyfriedelan-3-one	H	H	*α*-OH	*β*-OH	CH_3_	CH_3_	CH_3_	-	A6	[30]
**49**	3*β*,24*β*-epoxy-29-methoxy-2*α*,3*α*,6*α*-trihydroxy-D:A-friedelane	OH	OH	*β*-OH	-	-	-	-	-	A13	[31]
**49a**	3*β*,24*β*-epoxy-29-methoxy-2*α*,3*α*,6*α*-triacetoxy-D:A-friedelane	OAc	OAc	*β*-OAc	-	-	-	-	-	A13	[31]
**50**	Friedel-1-en-3,16-dione	-	-	-	-	-	-	-	-	A17	[32]
**51**	1*α*,29-dihydroxyfriedelan-3-one	*α*-OH	H	H	H	CH_3_	CH_3_	CH_2_OH	-	A6	[32]
**52**	16*β*,28,29-trihydroxyfriedelan-3-one	H	H	H	*β*-OH	CH_2_OH	CH_3_	CH_2_OH	-	A6	[32]
**53**	Dispemroquinone	=O	H	H	COOMe	-	-	-	-	A5	[33]
**54**	Scutione	H	=O	-	-	-	-	-	-	A10	[34]
**55**	Zeylasterone	OH	OH	COOH	COOMe	H	H	-	-	A2	[35]
**56**	Demethylzeylasterone	OH	OH	COOH	COOH	H	H	-	-	A2	[35]
**57**	3,15-dioxo-21-hydroxy friedelane	=O	H	=O	CH_3_	*α*-OH	-	-	-	A14	[36]
**58**	Maytenfoliol	H	H	H	H	CH_2_OH	CH_2_OH	CH_3_	-	A6	[37]
**59**	Cangorosin A	H	-	-	-	-	-	-	-	A18	[38]
**60**	Atropcangorosin A	H(atropisomer of 7-bu)	-	-	-	-	-	-	-	A18	[38]
**61**	Dihydroatropcangorosin A	6′,7′-dihydro derivative of 8-BU	-	-	-	-	-	-	-	A18	[38]
**62**	Cangorosin B	-	-	-	-	-	-	-	-	A19	[38]
**63**	Umbellatin *α*	*α*-Me	-	-	-	-	-	-	-	A20	[39]
**64**	Umbeilatin *β*	*β*-Me	-	-	-	-	-	-	-	A20	[39]
**65**	Tiscutin A	-	-	-	-	-	-	-	-	A21	[40]
**66**	Triscutin B	-	-	-	-	-	-	-	-	A22	[40]
**67**	Xuxuarine Eα	-	-	-	-	-	-	-	-	A23	[41]
**68**	Scutionin αB	-	-	-	-	-	-	-	-	A24	[42]
**69**	6′,7′-dihydro-scutionin αB	H	-	-	-	-	-	-	-	A25	[42]
**70**	6′*β*-methoxy-6′,7′dihydro-scutionin αB	OCH_3_	-	-	-	-	-	-	-	A25	[42]

**Table 2:** The lupane triterpenes isolated from *Maytenus*.

No.	Name	R_1_	R_2_	R_3_	R_4_	R_5_	R_6_	R_7_	R_8_	R_9_	Type	Ref
**71**	3*β*,28,30-Lup-20(29)-ene triol	H	H	OH	CH_3_	H	H	H	CH_2_OH	CH_2_OH	B1	[43]
**72**	28,30-Dihyroxylup-20(29)-ene-3-one	H	H	=O	CH_3_	H	H	H	CH_2_OH	CH_2_OH	B1	[43]
**73**	Maytefolins A	H	*α*-OH	=O	CH_3_	H	H	H	CH_2_OH	CH_3_	B1	[44]
**74**	3-oxolup-20(29)-en-30-al	H	H	=O	CH_3_	H	H	H	CH_3_	CHO	B1	[45]
**75**	30-hydroxylup-20(29)-en-3-one	H	H	=O	CH_3_	H	H	H	CH_3_	CH_2_OH	B1	[45]
**76**	(11*α*)-11-hydroxylup-20(29)-en-3-one	H	H	=O	CH_3_	H	*α*-OH	H	CH_3_	CH_3_	B1	[45]
**77**	(3*β*)-lup-20(30)-ene-3,29-diol	H	H	*β*-OH	CH_3_	H	H	H	CH_3_	CH_2_OH	B1	[45]
**78**	11*α*-hydroxy-*epi*-betuin	H	H	*α*-OH	CH_3_	H	*α*-OH	H	CH_2_OH	CH_3_	B1	[46]
**79**	6*β*-hydroxybetulin	H	H	*β*-OH	CH_3_	*β*-OH	H	H	CH_2_OH	CH_3_	B1	[46]
**80**	24-hydroxybetulin	H	H	=O	CH_2_OH	H	H	H	CH_2_OH	CH_3_	B1	[46]
**81**	Rigidenol-28-aldehyde	H	H	=O	CH_3_	H	*α*-OH	H	CHO	CH_3_	B1	[46]
**82**	28-hydroxyglochidone	H	CH_2_OH	-	-	-	-	-	-	-	B2	[46]
**83**	11*α*-hydroxy-glochidone	*α*-OH	CH_3_	-	-	-	-	-	-	-	B2	[47]
**84**	3-*epi-*nepeticin	H	H	*α*-OH	CH_3_	H	*α*-OH	H	CH_3_	CH_3_	B1	[47]
**85**	3-*epi-*calenduladiol	H	H	*α*-OH	CH_3_	H	H	H	OH	CH_3_	B1	[47]
**86**	3*α*,16*β*,28-Trihydroxylup-20(29)-ene	H	H	*α*-OH	CH_3_	H	H	*β*-OH	CH_2_OH	CH_3_	B1	[48]
**87**	3*α*,16*β*-dihydroxylup-12-ene	*α*-OH	*β*-OH	-	-	-	-	-	-	-	B3	[48]
**88**	3*β*,16*β*-dihydroxylup-12-ene	*β*-OH	*β*-OH	-	-	-	-	-	-	-	B3	[48]
**89**	16*β*-3,4-*seco*lup-20(29)-en-3-oic acid	*-*	-	-	-	-	-	-	-	-	B4	[48]
**90**	3-(*E*)-*β*-coumaroylnepeticin	H	H	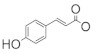	CH_3_	H	*α*-OH	H	CH_3_	CH_3_	B1	[25]
**91**	3,4-*seco*-lupa-4(23):20(29)-diene-3,28-dioicacid 28-methyl ester	-	-	-	-	-	-	-	-	-	B5	[26]
**92**	1*β*-Hydroxy-3*β*-caffeate lup-20(29)-ene	*β*-OH	H	OCaf	CH_3_	H	H	H	CH_3_	CH_3_	B1	[49]
**93**	3-oxo-21*β*-*H*-hop-22(29)-ene	=O	*-*	-	-	-	-	-	-	-	B6	[28]
**94**	3*β*-hydroxy-21*β*-*H*-hop-22(29)-ene	*β*-OH	*-*	-	-	-	-	-	-	-	B6	[28]
**95**	3,4-*seco*-21*β*-*H*-hop-22(29)-en-3-oic acid	-	-	-	-	-	-	-	-	-	B7	[28]

**Table 3:** The oleanane triterpenes isolated from *Maytenus*.

No.	Name	R_1_	R_2_	R_3_	R_4_	R_5_	R_6_	R_7_	R_8_	Type	Ref.
**96**	3*β*,19*α*-dihydroxyolean-12-en-29-oic acid	*β*-OH	H	H	CH_3_	H	*α*-OH	CH_3_	COOH	C1	[14]
**97**	3*α*,19*α*-dihydroxyolean-12-en-29-oic acid	*α*-OH	H	H	CH_3_	H	*α*-OH	CH_3_	COOH	C1	[14]
**98**	3-oxo-11*α*-methoxyolean-12-ene	=O	H	*α*-OCH_3_	CH_3_	H	H	CH_3_	CH_3_	C1	[24]
**99**	22*α*-hydroxy-29-methoxy-3*β*-tetradecanoate-olean-12-ene	*β*-COOC_13_H_27_	H	H	CH_3_	*α*-OH	H	CH_3_	COOMe	C1	[31]
**100**	Maytefolin B	-	-	-	-	-	-	-	-	C2	[44]
**101**	3*β*-peroxy-7*β*,25-epoxy-D:B-friedoolean-5-ene	-	-	-	-	-	-	-	-	C3	[48]
**102**	28-hydroxyolean-12-ene-3,11-dione	=O	H	=O	CH_2_OH	H	H	CH_3_	CH_3_	C1	[50]
**103**	6*β*,28-dihydroxyolean-12-ene-3,11-dione	=O	*β*-OH	=O	CH_2_OH	H	H	CH_3_	CH_3_	C1	[50]
**104**	3-oxo-11*α-*methoxyolean-12-ene-30-oic acid	=O	H	α-OCH_3_	CH_3_	H	H	COOH	CH_3_	C1	[51]
**105**	3-oxo-11*α*-hydroxyolean-12-ene-30-oic acid	=O	H	α-OH	CH_3_	H	H	COOH	CH_3_	C1	[51]
**106**	3-oxo-olean-9(11),12-diene-30-oic acid	=O	COOH	-	-	-	-	-	-	C4	[51]
**107**	3,4-*seco*-olean-4(23),12-diene-3,29-dioic acid	-	-	-	-	-	-	-	-	C5	[51]
**108**	3*α*-22*β*-dihydroxyolean-12-en-29-oicacid	*α*-OH	H	H	CH_3_	*β*-OH	H	CH_3_	COOH	C1	[52]
**109**	Olean-9 (11):12-dien-3*β*-ol	*β*-OH	CH_3_	-	-	-	-	-	-	C4	[53]
**110**	3*β*-Hydroxy-D:B-friedo-olean-5-ene	-	-	-	-	-	-	-	-	C6	[54]
**111**	19*α*-hydroxy-3-olean-12-en-29-oic acid	=O	H	H	CH_3_	H	*α*-OH	CH_3_	COOH	C1	[55]

**Table 4:** The other triterpenes isolated from *Maytenus*.

No.	Name	R_1_	R_2_	R_3_	R_4_	Type	Ref.
**112**	3-Oxo-methoxyurs-12-ene	=O	H	*α*-OCH_3_	CH_3_	D1	[24]
**113**	Krukovines B	=O	H	CH_2_OH	H	D1	[50]
**114**	Krukovines D	=O	OH	CH_2_OH	H	D1	[50]
**115**	Krukovines E	=O	H	OH	H	D1	[50]
**116**	Maytefolins C	-	-	-	-	D2	[44]
**117**	28-hydroxy-12-ursene-3*β*-yl-caffeate	*β*-OCaf	H	CH_2_OH	H	D1	[52]
**118**	3*β*-stearyloxy-urs-12-ene	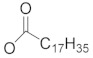	H	CH_3_	H	D1	[56]
**119**	24-(*E*)-3-oxo-dammara-20,24-dien-26-al	-	-	-	-	D3	[57]
**120**	24-(*Z*)-3-oxo-dammara-20,24-dien-26-al	-	-	-	-	D3	[57]
**121**	24-(*E*)-3-oxo-dammara-20,24-dien-26-ol	H	H	CH_3_	CH_2_OH	D4	[57]
**122**	24-(*E*)-3-oxo-dammara-23-*α*-hydroxy-20,24-dien-26-al	H	*α*-OH	CH_3_	CHO	D4	[57]
**123**	24-(*E*)-3-oxo-dammara-23-*β*-hydroxy-20,24-dien-26-al	H	*β*-OH	CH_3_	CHO	D4	[57]
**124**	24-(*E*)-3-oxo-dammara-6-*β*-hydroxy-20,24-dien-26-al	*β*-OH	H	CH_3_	CHO	D4	[57]
**125**	24-(*E*)-3-oxo-dammara-6-*β*-hydroxy-20,24-dien-26-ol	*β*-OH	H	CH_3_	CH_2_OH	D4	[57]
**126**	23-(*Z*)-3,25-dioxo-25-nor-dammara-20,24-diene	-	-	-	-	D5	[57]
**127**	24-(*E*)-3-oxo-23-methylene-dammara-20,24-dien-26-oico	-	-	-	-	D6	[58]
**128**	24(*Z*)-3-oxodammara20(21),24-dien-27-oic acid	H	H	COOH	CH_3_	D4	[58]
**129**	Octa-nor-13-hydroxydammara-1-en-3,17-dione	-	-	-	-	D7	[58]

**Table 5:** The sesqiterpenoids isolated from *Maytenus*.

No.	Name	R_1_	R_2_	R_3_	R_4_	R_5_	R_6_	R_7_	R_8_	Type	Ref.
**130**	1*α*,9*α*-dibenzoyloxy-6*β*,8*α*,15-triacetoxy-4*β*-hydroxy-dihydro-*β*-agarofurane	OBz	H	H	OAc	CH_3_	*α*-OAc	*α*-OBz	CH_2_OAc	E1	[60]
**131**	1*α*,9*α*-dibenzoyloxy-2*α*,6*β*,8*α*,15-tetracetoxy-4*β*-hydroxydihydro-*β*-agrofurane	OBz	*α*-OAc	H	OAc	CH_3_	*α*-OAc	*α*-OBz	CH_2_OAc	E1	[60]
**132**	6*β*,8*β*-15-triacetoxy-1*α*,9*α*-dibenzoyloxy-4*β*-hydroxy-*β*-dihydroagarofuran	OBz	H	H	OAc	CH_3_	*β*-OAc	*α*-OBz	CH_2_OAc	E1	[61]
**133**	1*α*,6*β*,8*β*-15-tetraacetoxy-9*α*-benzoyloxy-4*β*-hydroxy-*β*-dihydroagarofuran	OAc	H	H	OAc	CH_3_	*β*-OAc	*α*-OBz	CH_2_OAc	E1	[61]
**134**	(1*S*,4*S*,6*R*,7*S*,8*S*,9*R*)-1,6,15- triacetoxy-8,9-dibenzoyloxy-4*β*-hydroxy-*β*-dihydroagarofuran	OAc	H	H	OAc	CH_3_	*α*-OBz	*β*-OBz	CH_2_OAc	E1	[61]
**135**	(1*R*,2*S*,4*S*,5*S*,6*R*,7*R*,9*S*,10*S*)-6,15-diacetoxy-1,2,9-tribenzoyloxy-4-hrdroxy-8-oxo-dihydro-*β*-agarofuran	OBz	*α*-OBz	H	OAc	CH_3_	O	*α*-OBz	CH_2_OAc	E1	[41]
**136**	9*β*-cinnamoyloxy-2*β*,3*β*-diacetoxy-6*β*-hydroxy-l*α*-nicotinoyloxydihidro-*β*-agarofuran	ONic	*β*-OAc	*β*-OAc	OH	CH_3_	H	*β*-OCin	CH_3_	E1	[41]
**137**	1*α*-acetoxy-2*α*,6*β*,9*β*-trtifuroyloxy-4*β*-hydroxy-dihydro-*β*- agarofuran	OAc	*α*-OFu	H	OFu	CH_3_	H	*β*-OFu	CH_3_	E1	[59]
**138**	1*α*,2*α*-diacetoxy-6*β*,9*β*-difuroyloxy-4*β*-hydroxy-dihydro-*β*- agarofuran	OAc	*α*-OAc	H	OFu	CH_3_	H	*β*-OFu	CH_3_	E1	[59]
**139**	1*α*-acetoxy-6*β*,9*β*-difuroyloxy-2*α*,4*β*-dihydroxy-dihydro-*β*-agarofuran	OAc	*α*-OH	H	OFu	CH_3_	H	*β*-OFu	CH_3_	E1	[59]
**140**	1*α*-acetoxy-2*α*-benzoyloxy-6*β*,9*β*-difuroyloxy-4*β*-dihydro-*β*-agarofuran	OAc	*α*-OBz	H	OFu	CH_3_	H	*β*-OFu	CH_3_	E1	[59]
**141**	1*α*-acetoxy-6*β*,9*β*-difuroyloxy-2*α*-propyonyloxy-4*β*-hydroxy-dihydro-*β*-agarofuran	OAc	*α*-OPr	H	OFu	CH_3_	H	*β*-OFu	CH_3_	E1	[59]
**142**	1*α*-acetoxy-6*α*,9*β*-difuroyloxy-2*α*-(2)-methylbutyroyloxy-4*β*-hydroxy-dihydro-*β*-agarofuran	OAc	*α*-OButMe	H	OFu	CH_3_	H	*β*-OFu	CH_3_	E1	[59]
**143**	1*α*, 2*α*,15-triacetoxy-6*β*,9*β*-difuroyloxy-4*β*- hydroxy-dihydro-*β*-agarofuran	OAc	*α*-OAc	H	OFu	CH_3_	H	*β*-OFu	CH_2_OAc	E1	[59]
**144**	1α, 2α,15-triacetoxy-6β,9β-dibenzoyloxy-4β- hydroxy-dihydro-*β*-agarofuran	OAc	*α*-OAc	H	OBz	CH_3_	H	*β*-OBz	CH_2_OAc	E1	[59]
**145**	(1*R*,2*R*,4*S*,5*R*,7*S*,9*S*,10*R*)-2-acetoxy-1-benzoyloxy-9-cinnamoyloxy-4-hydroxy- dihydro-*β*-agarofuran	OBz	*β*-OAc	H	H	CH_3_	H	*β-*OH	CH_3_	E1	[62]
**146**	(1*R*,2*S*,3*S*,5*R*,7*R*,9*S*,10*R*)-2-acetoxy-9-benzoyloxy-1-cinnamoyloxy-3-nicotinoyloxy-4-hydroxy-dihydro-*β*-agarofuran	OCin	*β*-OAc	*β*-ONic	H	CH_3_	H	H	CH_3_	E1	[62]
**147**	(1*R*,2*S*,3*S*,4*S*,5*S*,6*R*,7*R*,9*S*,10*R*)-2,6-diacetoxy-1-benzoyloxy-9-cinnamoyloxy-3-nicotinoyloxy-4-hydroxy-dihydro-*β*-agarofuran	OBz	*β*-OAc	*β*-ONic	OAc	CH_3_	H	*β-*OCin	CH_3_	E1	[62]
**148**	(1*R*,2*S*,3*S*,4*S*,5*S*,6*R*,7*R*,9*S*,10*R*)-2,6-diacetoxy-1,9-dibenzoyloxy-3-nicotinoyloxy-4-hydroxy-dihydro-*β*-agarofuran	OBz	*β*-OAc	*β*-ONic	H	CH_3_	H	*β*-OBz	CH_3_	E1	[62]
**149**	(1*R*,2*S*,3*S*,4*S*,5*R*,7*S*,8*S*,9*R*,10*R*)-2,3-diacetoxy-8,9-dibenzoyloxy-1-nicotinoyloxy-4-hydroxy-dihydro-*β*-agarofuran	ONic	*β*-OAc	*β*-OAc	H	CH_3_	*β*-OBz	*β*-OBz	CH_3_	E1	[62]
**150**	(1*R*,2*S*,4*S*,5*S*,6*R*,7*R*,8*S*,9*R*,10*S*)-6,8-diacetoxy-1,2,9-tribenzoyloxy-4-hydroxy-dihydro-*β*-agarofuran	OBz	*α*-OBz	H	OAc	CH_3_	*β*-OAc	*β*-OBz	CH_3_	E1	[62]
**151**	(1*R*,2*S*,3*S*,4*S*,5*R*,7*S*,8*S*,9*R*,10*R*)-2,8-diacetoxy-3,9-dibenzoyloxy-1-nicotinoyloxy-4-hydroxy-dihydro-*β*-agarofuran	ONic	*β*-OAc	*β*-OBz	H	*β*-OAc	*β*-OBz	CH_3_	-	E2	[62]
**152**	(1*R*,2*S*,4*R*,5*S*,6*R*,7*R*,8*S*,9*R*,10*S*)-6,8-diacetoxy-1,9-dibenzoyloxy-2-nicotinoyloxy-dihydro-*β*-agarofuran	OBz	*α*-ONic	H	OAc	*β*-OAc	*β*-OBz	CH_3_	-	E2	[62]
**153**	1*α*,15-diacetoxy-6*β*,9*β*-dibenzoyloxy-2*α*-nicotinoyloxy-dihydro-*β*-agarofuran	OAc	*α*-ONic	H	OBz	H	*β*-OBz	CH_3_	-	E2	[62]
**154**	1*α*,15-diacetoxy-6*β*,9*β*-dibenzoyloxy-2*α*-nicotinoyloxy-4*β*-hydroxy-dihydro-*β*-agarofuran	OAc	*α*-ONic	H	OBz	CH_3_	H	*β*-OBz	CH_2_OAc	E1	[62]
**155**	(1*R*,2*S*,4*S*,5*S*,6*R*,7*R*,9*S*,10*S*)-1,2,6,9,15-pentaacetoxy-4-hydroxy-8-oxo-dihydro-*β*-agarofuran	OAc	*α*-OAc	H	OAc	CH_3_	O	*α*-OAc	CH_2_OAc	E1	[63]
**156**	(1*R*,2*S*,4*S*,5*S*,6*R*,7*R*,9*S*,10*S*)-1,2,9,15-taacetoxy-4,6-dihydroxy-8-oxo-dihydro-*β*-agarofuran	OAc	*α*-OAc	H	OH	CH_3_	O	*α*-OAc	CH_2_OAc	E1	[63]
**157**	(1*R*,2*S*,4*S*,5*S*,6*R*,7*R*,9*S*,10*S*)-1,9,15-triacetoxy-2,4,6-trihydroxy-8-oxo-dihydro-*β*-agarofuran	OAc	*α*-OH	H	OH	CH_3_	O	*α*-OAc	CH_2_OAc	E1	[63]
**158**	(1*R*,2*S*,3*S*,4*S*,5*S*,6*R*,7*R*,9*S*,10*S*)-1,2,3,6,9,12,15-heptaacetoxy-4-hydroxy-8-oxo-dihydro-*β*-agarofuran	OAc	*α*-OAc	*β*-OAc	OAc	CH_2_OAc	O	*α*-OAc	CH_2_OAc	E1	[63]
**159**	1*α*,2*α*,3*β*,6*β*,8*α*,9*α*,12,15-octaacetoxy-4*β*-hydroxy-dihydro-*β*-agarofuran	OAc	*α*-OAc	*β*-OAc	OAc	CH_2_OAc	*α*-OAc	*α*-OAc	CH_2_OAc	E1	[63]
**160**	(1*S*,4*S*,5*S*,6*R*,7*R*,8*S*,9*R*,10*R*)-8-acetoxy-1,9-dibenzoyloxy-6-nicotynoyloxy-dihydro-*β*-agarofuran	OBz	H	H	ONic	*α*-OAc	*α*-OBz	CH_3_	-	E2	[49]
**161**	(1*S*,4*R*,5*R*,6*R*,7*R*,8*S*,9*R*,10*R*)-8-acetoxy-1,9-dibenzoyloxy-4-hydroxy-nicotynoyloxy-dihydro-*β*-agarofuran	OBz	H	H	ONic	CH_3_	*α*-OAc	*α*-OBz	CH_3_	E1	[49]
**162**	(1*R*,2*S*,4*S*,5*S*,6*R*,7*R*,9*S*,10*R*)-1,15-diacetoxy-2,6-dibenzoyloxy-9-(3-furoyloxy)-4-hydroxy-dihydro-*β*-agarofuran	OAc	*α*-OBz	H	OBz	CH_3_	H	*α*-OFu	CH_2_OAc	E1	[64]
**163**	(1*R*,2*S*,4*S*,5*S*,6*R*,7*R*,9*S*,10*R*)-1,2,15-triacetoxy-6-benzoyloxy-9-(3-furoyloxy)-4-hydroxy-dihydro-*β*-agarofuran	OAc	*α*-OAc	H	OBz	CH_3_	H	*α*-OFu	CH_2_OAc	E1	[64]
**164**	(1*R*,2*S*,4*S*,5*S*,6*R*,7*R*,9*S*,10*R*)-1,15-diacetoxy-6-benzoyloxy-9-(3-furoyloxy)-2,4-dihydroxy-dihydro-*β*-agarofuran	OAc	*α*-OH	H	OBz	CH_3_	H	*α*-OFu	CH_2_OAc	E1	[64]
**165**	(1*R*,2*S*,4*S*,5*S*,6*R*,7*R*,9*S*,10*R*)-1,15-diacetoxy-6,9-dibenzoyloxy-2,4-hydroxy-dihydro-*β*-agarofuran	OAc	*α*-OH	H	OBz	CH_3_	H	*α*-OBz	CH_2_OAc	E1	[64]
**166**	(1*R*,2*S*,4*S*,5*S*,6*R*,7*R*,9*S*,10*R*)-1,2,6,15-tetracetoxy-9-(3-furoyloxy)-4-hydroxy-dihydro-*β*-agarofuran	OAc	*α*-OAc	H	OAc	CH_3_	H	*α*-OFu	CH_2_OAc	E1	[64]
**167**	(1*R*,2*S*,4*S*,5*S*,6*R*,7*R*,9*S*,10*R*)-1-Acetoxy-2,6-dibenzoyloxy-9-(3-furoyloxy)-4-hydroxy-dihydro-*β*-agarofuran	OAc	*α*-OBz	H	OBz	CH_3_	H	*α*-OFu	CH_3_	E1	[64]
**168**	(1*S*,2*S*,3*S*,4*S*,5*R*,7*R*,9*S*,10*R*)-2,3-diacetoxy-9-benzoyloxy-1-(3-furoyloxy)-4-hydroxy-dihydro-*β*-agarofuran	OFu	*β*-OAc	*β*-OAc	H	CH_3_	H	*α*-OBz	CH_3_	E1	[64]
**169**	(1*S*,2*R*,4*S*,5*R*,7*R*,9*S*,10*R*)-2-acetoxy-9-benzoyloxy-1-(3-furoyloxy)-4-hydroxy-dihydro-*β*-agarofuran	OFu	*β*-OAc	H	H	CH_3_	H	*α*-OBz	CH_3_	E1	[64]
**170**	(1*S*,2*R*,4*S*,5*R*,7*R*,9*S*,10*R*)-2-Acetoxy-1,9-di-(3-furoyloxy)-4-hydroxy-dihydro-*β*-agarofuran	OFu	*β*-OAc	H	H	CH_3_	H	*α*-OFu	CH_3_	E1	[64]
**171**	(1*S*,2*R*,4*S*,5*R*,7*R*,9*S*,10*R*)-2-Acetoxy-9-trans-cynamoiloxy-1-(3-furoyloxy)-4-hydroxy-dihydro-*β*-agarofuran	OFu	*β*-OAc	H	H	CH_3_	H	*α*-OCin	CH_3_	E1	[64]
**172**	(1*S*,4*S*,5*R*,7*R*,9*S*,10*S*)-9-Benzoyloxy-1-(3-furoyloxy)-4-hydroxy-dihydro-*β*-agarofuran	OFu	H	H	H	CH_3_	H	*α*-OBz	CH_3_	E1	[64]
**173**	(1*S*,2*R*,3*R*,4*R*,5*S*,7*R*,9*S*,10*R*)-2,3-diacetoxy-9-benzoyloxy-1-(3-furoyloxy)-dihydro-*β*-agarofuran	OFu	*β*-OAc	*β*-OAc	H	H	*α*-OBz	CH_3_	-	E2	[64]
**174**	(1*S*,2*R*,4*R*,5*S*,7*R*,9*S*,10*R*)-2-Acetoxy-9-benzoyloxy-1-(3-furoyloxy)-dihydro-*β*-agarofuran	OFu	*β*-OAc	H	H	H	*α*-OBz	CH_3_	-	E2	[64]
**175**	1*α*,2*α*,9*β*,15-tetracetoxy-8*β*-benzoyloxy-*β*-dihydroagarofuran	OAc	*α*-OAc	H	H	*β*-OBz	*α*-OAc	CH_2_OAc	-	E2	[65]
**176**	1*α*-benzoyloxy-2*α*,6*β*,8*α*-triacetoxy-9α-methyllbutyroyloxy-*β*-dihydroagarofuran	OBz	*α*-OAc	H	OAc	*α*-OAc	*α*-OMeBut	CH_3_	-	E2	[65]
**177**	1*α*,6*β*-diacetoxy-2*α*,8*α*,9*α*-tribenzoyloxy-*β*-dihydroagarofuran	OAc	*α*-OBz	H	OAc	*α*-OBz	*α*-OBz	CH_3_	-	E2	[65]
**178**	1*α*-benzoyloxy-2*α*,6*β*,8*α*,9*α*-tetraacetoxy-*β*-dihydroagarofuran	OBz	*α*-OAc	H	OAc	*α*-OAc	*α*-OAc	CH_3_	-	E2	[65]
**179**	1*α*,6*β*,8*α*-triacetoxy-9*α*-benzoyloxy-2*α*-hydroxy-*β*-dihydroagarofuran	OAc	*α*-OH	H	OAc	*α*-OAc	*α*-OBz	CH_3_	-	E2	[65]
**180**	(1*R*,2*S*,4*R*,5*S*,6*R*,7*R*,8*R*,9*S*,10*S*)-1,6-diacetoxy-8,9-dibenzoyloxy-2-h ydroxy-*β*-d ihydroagarofuran	OAc	*α*-OH	H	OAc	*α*-OBz	*α*-OBz	CH_3_	-	E2	[65]
**181**	1*α*,6*β*,15-triacetoxy-8*α*-methylbutyroyloxy-9*α*-benzoyloxy-2*α*-hydroxy-*β*-dihydroagaro-furan	OAc	*α*-OH	H	OAc	*α*-OMeBut	*α*-OBz	CH_2_OAc	-	E2	[65]
**182**	1*α*,6*β*,1*5*-triacetoxy-8*α*,9*α*-dibenzoyloxy-2*α*-hydroxy-*β*-dihydroagarofuran	OAc	*α*-OH	H	OAc	*α*-OBz	*α*-OBz	CH_2_OAc	-	E2	[65]
**183**	1*α*,6*β*,8*β*,15-tetracetoxy-2*α*-hydroxy-9*α*-benzoyloxy-*β*-dihydroagarofuran	OAc	*α*-OH	H	OAc	*β*-OAc	*α*-OBz	CH_2_OAc	-	E2	[65]
**184**	Chiapens A	OH	*α*-OAc	H	OAc	*α*-OBz	*α*-OBz	CH_2_OAc	-	E2	[66]
**185**	Chiapens B	OAc	*α*-OAc	H	OAc	*α*-OBz	*α*-OBz	CH_2_OAc	-	E2	[66]
**186**	Chiapens C	OH	H	H	OAc	*α*-OBz	*α*-OBz	CH_2_OAc	-	E2	[66]
**187**	Chiapens D	OAc	*α*-OAc	H	OBut	H	*α*-OBz	CH_2_OAc	-	E2	[66]
**188**	Chiapens E	OAc	*α*-OAc	*β*-OAc	OAc	O	*α*-OBz	CH_2_OAc	-	E2	[66]
**189**	1*α*,6*β*-diacetoxy-8*α*-hydroxy-9*β*-furoyloxy-*β*-agarofuran	OAc	H	H	OAc	*α*-OH	*β*-OFu	CH_3_	-	E2	[67]
**190**	1*α*-acetoxy-6*β*,8*α*-dihydroxy-9*β*-furoyloxy-*β*-agarofuran	OAc	H	H	OH	*α*-OH	*β*-OFu	CH_3_	-	E2	[67]
**191**	1*α*-benzoyloxy-2*α*,3*β*,6*β*,9*β*,14-pentaacetoxy-8-oxo-*β*-agarofuan	OBz	*α*-OAc	*β*-OAc	OBz	O	OAc	CH_2_OAc	-	E2	[67]
**192**	1*α*-furoyloxy-2*α*,3*β*,6*β*,9*β*,14-pentaacetoxy-8-oxo-*β*-agarofuan	OFu	*α*-OAc	*β*-OAc	OFu	O	OAc	CH_2_OAc	-	E2	[67]
**193**	Bilocularins A	OAc	H	H	OAc	*α*-OH	*α*-OBz	CH_2_OAc	-	E2	[68]
**194**	Bilocularins B	OAc	H	H	OH	*α*-OAc	*α*-OBz	CH_2_OAc	-	E2	[68]
**195**	Bilocularins C	OAc	H	H	OAc	O	*α*-OBz	CH_2_OAc	-	E2	[68]
**196**	Bilocularins D	OHAc	*α*-OAc	H	OAc	CH_3_	H	*β*-O*t*Cin	CH_2_OBz	E1	[69]
**197**	Bilocularins E	OHAc	*α*-OAc	H	OAc	CH_3_	H	*β*-O*t*Cin	CH_2_O*t*Cin	E1	[69]
**198**	Bilocularins F	OHAc	*α*-OAc	H	OAc	CH_3_	H	*β*-O*t*Cin	CH_2_OH	E1	[69]
**199**	Bilocularins G	OAc	*α*-OAc	H	OAc	CH_3_	H	*β*-O*t*Cin	CH_2_OAc	E1	[69]
**200**	Bilocularins H	OAc	*α*-OH	H	OAc	CH_3_	H	*β*-O*t*Cin	CH_2_OH	E1	[69]
**201**	Bilocularins I	OAc	*α*-OH	H	ONic	CH_3_	H	*β*-O*t*Cin	CH_3_	E1	[69]
**202**	(1*S*,4*S*,5*S*,6*R*,7*R*,8*R*,9*R*,10*S*)-6-acetoxy-4,9,10-trihydroxy-2,2,5*a*,9-tetramethyloctahydro-2*H*-3,9*a*-methanobenzo[*b*]-oxepin-5-yl furan-3-carboxylate	OAc	H	H	OH	CH_3_	*α*-OH	*β*-OFu	CH_3_	E1	[70]
**203**	(1*S*,4*S*,5*S*,6*R*,7*R*,8*R*,9*R*,10*S*)-6-acetoxy-4,9-dihydroxy-2,2,5*a*,9-tetramethyloctahydro-2*H*-3,9a-methanobenzo[*b*]oxepine-5,10-diylbis(furan-3-carboxylate)	OAc	H	H	OFu	CH_3_	*α*-OH	*β*-OFu	CH_3_	E1	[71]
**204**	(1*S*,4*S*,5*S*,6*R*,7*R*,9*S*,10*S*)-6-acetoxy-9-hydroxy-2,2,5*a*,9-tetramethyloctahydro-2*H*-3,9*a*-methanobenzo[*b*]oxepine-5,10-diyl bis(furan-3-carboxylate)	OAc	H	H	OFu	CH_3_	H	*β*-OFu	CH_3_	E1	[71]
**205**	(1*S*,4*S*,5*S*,6*R*,7*R*,9*S*,10*S*)-6-acetoxy-10-(benzoyloxy)-9-hydroxy-2,2,5*a*,9-tetramethyloctahydro-2*H*-3,9*a*-methanobenzo[*b*]-oxepin-5-yl furan-3-carboxylate	OAc	H	H	OBz	CH_3_	H	*β*-OFu	CH_3_	E1	[71]
**206**	2*β*,6*β*-diacetoxy-1*α*,9*β*-dibenzoyl-3*β*-hydroxy-dihydro-*β*-agarofuran	*α*-OBz	*β*-OAc	*β*-OH	H	*β*-CH_3_	-	-	-	E3	[72]
**207**	1*α*,2*α*,6*β*,8*α*-tetraacetoxy-9*β*-benzoyl-15-hydroxy-dihydro-*β*-agarofuran	*α*-OAc	*α*-OAc	H	*α*-OAc	*α*-CH_2_OH	-	-	-	E3	[72]
**208**	1*α*,2*α*,6*β*,8*α*,1*5*-pentaacetoxy-9*β*-benzoyl-dihydro-*β*-agarofuran	*α*-OAc	*α*-OAc	H	*α*-OAc	*α*-CH_2_OAc	-	-	-	E3	[72]
**209**	1*β*-acetoxy-9*α*-benzoyloxy-2*β*,6*α*-dinicotinoyioxy-*β*-dihydro-agarofuran(heterophylline)	-	-	-	-	-	-	-	-	E4	[73]
**210**	Boarioside	-	-	-	-	-	-	-	-	E5	[74]
**211**	4-deacetyl-10-oxo-dihydrobotrydial	-	-	-	-	-	-	-	-	E6	[75]
**212**	4*β*-acetoxy-9*β*,10*β*,15*α*-trihydroxyp robotrydial	-	-	-	-	-	-	-	-	E7	[75]

**Table 6:** The sesquiterpene pyridine alkaloids isolated from *Maytenus*.

No.	Name	R_1_	R_2_	R_3_	R_4_	R_5_	Type	Ref.
**213**	Wilforine	-	-	-	-	-	F1	[77]
**214**	Emarginatine-A	OAc	OAc	OAc	H	OAc	F2	[78]
**215**	Emarginatine-B	OAc	Benzoate	H	OAc	OAc	F2	[78]
**216**	Emarginatine-C	OAc	OH	OAc	H	OAc	F2	[79]
**217**	Emarginatine-D	OA	OAc	OAc	H	OAc	F2	[79]
**218**	Emarginatine-E	OH	OH	H	OAc	OAc	F2	[79]
**219**	Emarginatine-F	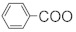	OAc	H	OH	OAc	F2	[11]
**220**	Emarginatine-G	CH_3_CH=CCH_3_OO	OAc	OAc	H	OAc	F2	[11]
**221**	Emarginatine-H	OAc	OAc	OAc	H	OH	F2	[22]
**222**	Emarginatinine	-	-	-	-	-	F3	[79]
**223**	Ebenifoline W-I	*β*-OAc	OBz	OBz	OAc	-	F4	[80]
**224**	Ebenifoline E-I	*β*-OAc	OAc	OH	OBz	OAc	F5	[80]
**225**	Ebenifoline E-II	*β*-OAc	OBz	OAc	OBz	OAc	F5	[80]
**226**	Aquifoliunine E-I	*α*-OBz	OAc	OAc	OAc	CH_2_OAc	F5	[81]
**227**	Aquifoliunine E-II	*α*-OH	OAc	OH	OAc	CH_2_OAc	F5	[81]
**228**	Aquifoliunine E-III	*α*-OH	OAc	OAc	OAc	CH_2_OAc	F5	[82]
**229**	Aquifoliunine E-IV	*α*-ONic	OAc	OAc	OAc	CH_2_OAc	F5	[82]
**230**	Ilicifoliunines A	*α*-OBz	OH	OAc	OAc	CH_2_OAc	F5	[83]
**231**	Ilicifoliunines B	*α*-OBz	OAc	OAc	CH_2_OAc	-	F4	[83]
**232**	Mayteine	*β*-OAc	OAc	OAc	OBz	CH_2_OAc	F5	[83]
**233**	Laevisines A	*β*-OAc	OAc	OAc	OCOC(CH_3_)=CHCH_3_	CH_2_OAc	F5	[84]
**234**	Laevisines B	β-OAc	OAc	ONic	CH_2_OAc	-	F4	[84]
**235**	Mekongensine	Ac	H	OAc	OAc	-	F6	[85]
**236**	7-*epi*-mekongensine	Ac	OAc	H	OAc	-	F6	[85]
**237**	1-*O*-benzoyl-1-deacetylmekongensine	Bz	H	OAc	OAc	-	F6	[85]
**238**	9′-deacetoxymekongensine	Ac	H	OAc	H	-	F6	[85]
**239**	1-*O*-benzoyl-1-deacetyl-9′-deacetoxymekongensine	Bz	H	OAc	H	-	F6	[85]
**240**	7-*epi*-euojaponine	Bz	Ac	H	OAc	H	F7	[85]
**241**	2-*O*-benzoyl-2-deacetylmayteine	Bz	Bz	Ac	H	OAc	F7	[85]
**242**	7-*epi*-5-*O*-benzoyl-5-deacetylperitassine A	-	-	-	-	-	F8	[85]
**243**	5-benzoyl-5-deacetylwilforidine	-	-	-	-	-	F9	[86]
**244**	Putterines A	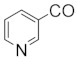	OAc	COOCH_3_	COOCH_3_	CH_2_OAc	F5	[76]
**245**	Putterines B	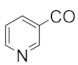	OAc	COOCH(CH_3_)_2_	COOCH_3_	CH_2_OAc	F5	[76]
**246**	7-(acetyloxy)-*O*^11^-benzoyl-*O*^2,11^-deacetyl-7-deoxoevonine	*β*-OAc	OAc	OH	OAc	CH2OBz	F5	[52]
**247**	Chiapenines ES-I	OBz	OBz	*α*-OAc	-	-	F10	[87]
**248**	Chiapenines ES-II	OBz	OBz	=O	-	-	F10	[87]
**249**	Chiapenines ES-III	OBz	OH	=O	-	-	F10	[87]
**250**	Chiapenines ES-IV	OAc	OH	=O	-	-	F10	[87]
**251**	Jelskiine	OiBut	*α*-OAc	OH	OAc	-	F11	[88]
**252**	*O*^9^-benzoyl-*O*^9^-deacetylevonine	OBz	=O	OAc	OAc	-	F11	[24]
**253**	8*β*-acetoxy-*O*^1^-benzoyl- *O*^1^-deacetyl-8-deoxoevonine	OAc	*β*-OAc	OAc	OBz	-	F11	[24]
**254**	1*α*,2*α*,6*β*,8*β*,9*α*,15-hexacetoxy-4*β*-hydroxy-3*β*,13-[2′-(3-carboxybutyl)]nicotinicacid-dicarbo-lactone-*β*-dihydroagarofuran	-	-	-	-	-	F12	[65]
**255**	1*α*,2*α*,9*α*,15-tetracetoxy-4*β*,6*β*-dihydroxy-8-oxo,3*β*,13-[4′-(3-carboxybutyl)]nicotinicacid-dicarbolactone-*β*-dihydroagarofuran	=O	-	-	-	-	F13	[65]
**256**	1*α*,2*α*,9*α*,15-tetracetoxy-4*β*,6*β*,8*β*-trihydroxy-3*β*,13-[4′-(3-carboxybutyl)]nicotinicacid-dicarbolactone-*β*-dihydroagarofuran	*β*-OH	-	-	-	-	F13	[65]
**257**	1*α*,2*α*,8*β*,9*α*,15-pent acetoxy-4*β*,6*β*-dihydroxy-3*β*,13-[4′-(3-carboxybutyl)]nicotinicaciddicarbolactne-*β*-dihydroagarofuran	*β*-OAc	-	-	-	-	F13	[65]
**258**	4-deoxyalatamine	OAc	OAc	-	-	-	F14	[31]
**259**	1-*O*-benzoyl-1-deacetyl-4-deoxy-alatamine	OBz	OAc	-	-	-	F14	[31]
**260**	1,2-*O*-dibenzoyl-1,2-deacetyl-4-deoxyalatamine	OBz	OBz	-	-	-	F14	[31]
**261**	4-deoxyisowilfordine	-	-	-	-	-	F15	[31]

## Data Availability

Not applicable.

## Sample Availability

Samples of the compounds are not available from the authors.
